# Genomic landscape of oxidative DNA damage and repair reveals regioselective protection from mutagenesis

**DOI:** 10.1186/s13059-018-1582-2

**Published:** 2018-12-07

**Authors:** Anna R. Poetsch, Simon J. Boulton, Nicholas M. Luscombe

**Affiliations:** 1 The Francis Crick Institute, 1 Midland Road, London, NW1 1AT UK; 2 Okinawa Institute of Science and Technology Graduate University, Okinawa, 904-0495 Japan; 3 UCL Genetics Institute, University College London, Gower Street, London, WC1E 6BT UK

## Abstract

**Background:**

DNA is subject to constant chemical modification and damage, which eventually results in variable mutation rates throughout the genome. Although detailed molecular mechanisms of DNA damage and repair are well understood, damage impact and execution of repair across a genome remain poorly defined.

**Results:**

To bridge the gap between our understanding of DNA repair and mutation distributions, we developed a novel method, AP-seq, capable of mapping apurinic sites and 8-oxo-7,8-dihydroguanine bases at approximately 250-bp resolution on a genome-wide scale. We directly demonstrate that the accumulation rate of apurinic sites varies widely across the genome, with hot spots acquiring many times more damage than cold spots. Unlike single nucleotide variants (SNVs) in cancers, damage burden correlates with marks for open chromatin notably H3K9ac and H3K4me2. Apurinic sites and oxidative damage are also highly enriched in transposable elements and other repetitive sequences. In contrast, we observe a reduction at chromatin loop anchors with increased damage load towards inactive compartments. Less damage is found at promoters, exons, and termination sites, but not introns, in a seemingly transcription-independent but GC content-dependent manner. Leveraging cancer genomic data, we also find locally reduced SNV rates in promoters, coding sequence, and other functional elements.

**Conclusions:**

Our study reveals that oxidative DNA damage accumulation and repair differ strongly across the genome, but culminate in a previously unappreciated mechanism that safeguards the regulatory and coding regions of genes from mutations.

**Electronic supplementary material:**

The online version of this article (10.1186/s13059-018-1582-2) contains supplementary material, which is available to authorized users.

## Introduction

The integrity of DNA is constantly challenged by damaging agents and chemical modifications. Base oxidation is a frequent insult that can arise from endogenous metabolic processes as well as from exogenous sources such as ionizing radiation. At background levels, a human cell is estimated to undergo 100 to 500 such modifications per day, most commonly resulting in 8-oxo-7,8-dihydroguanine (8-oxoG) and related products [1], which are then processed into repair intermediates. At steady state, up to 2400 8-oxoG sites per cell are reported [2]. However, estimates differ widely due to differences in methodology [3–10].

Oxidative damage is processed in a two-step process through the base excision repair (BER) pathway [11]. The damaged base is first recognized and excised by 8-oxoguanine DNA glycosylase 1 (OGG1), leaving an apurinic site (AP-site). Glycohydrolysis is highly efficient, with an 8-oxoG half-life of 11 min [12]. AP-sites are removed through backbone incision by AP-lyase (APEX1), and end processing through flap-endonuclease 1 (FEN1), and the base is subsequently replaced with an undamaged nucleotide. Alternatively, in short-patch base excision repair, replacement is dependent on polymerase beta. Other sources of AP-sites include spontaneous depurination and excision of non-oxidative base modifications, such as uracil. Cells are reported to typically present with a steady state of ~ 15,000 to ~ 30,000 AP-sites per cell, which includes the associated beta-elimination product [2, 13]. Left unrepaired, 8-oxoG can compromise transcription [5–7], DNA replication [8], and telomere maintenance [9]. Also, AP-sites can lead to genomic instability and compromise genomic processes [14]. Moreover, damaged sites provide direct and indirect routes to C-to-A mutagenesis [10, 15, 16].

Ionizing radiation is one of the most relevant exogenous sources of high-level oxidative DNA damage and DNA strand breaks. Each gray (Gy) is estimated to lead to ~ 10^6^ ionization events in the nucleus, only ~ 2000 of which are supposed to target DNA directly [17]. Most DNA damage from ionizing radiation occurs indirectly from radiolysed water and 60–70% can be prevented through radical scavenging [18, 19]. While absolute numbers differ throughout the literature, Lehnert estimates 1000–2000 base modifications per gray, 250 alkali labile sites, 1000 single-strand breaks (SSB), and 40 double-strand breaks. Others report base modifications to be threefold more prevalent than SSBs [20] or even several orders of magnitude increased [21, 22]. Interestingly, direct formation of AP-sites however has been shown not to increase more than 5% from background levels [23]. Therefore, after ionizing radiation, most AP-sites likely arise from excision of oxidized bases, which comprise mostly of 8-oxoG and the related modification FaPy-guanine [24].

Though originally controversial [25, 26], there is now broad acceptance that mutation rates vary across different genomic regions. Background mutation rates in *Escherichia coli* were shown to vary non-randomly between genes by an order of magnitude, with highly expressed genes displaying lower mutation rates [27]. In cancer genomes, single nucleotide variants (SNVs) tend to accumulate preferentially in heterochromatin [28, 29]. More recently, it was reported that SNV densities in cancers are lower in regions surrounding transcription factor binding but are elevated at the binding sites themselves and at sites with high nucleosome occupancy [30–33]. These variabilities likely arise through a combination of regional differences in damage sensitivity and the accessibility to the DNA repair machinery [34]. However, since mutations represent the endpoint of mutagenesis, it is impossible to tease apart the contributions from damage and repair through re-sequencing alone.

The role of oxidative damage in regional differences of mutagenesis remains largely unclear. Repair intermediates remain unexplored, but the genome-wide distribution of 8-oxoG has been studied through chemical enrichment [35–37] and immunoprecipitation [35–39]. The specificity of 8-oxoG antibodies, however, remains questionable [36, 40, 41], and the studies using chemical enrichment also arrive at disparate conclusions. Both Wu et al. [37] and Ding et al. [36] find 8-oxoG enriched at telomeres in yeast and mouse embryonic fibroblasts, respectively. However, Wu et al. find 8-oxoG largely depleted at promoters, while Ding et al. report increased damage at these sites. Therefore, we reassessed the raw data and did not find evidence for increased 8-oxoG at promoters (Additional file 1: Figure S6). Using antibodies, however, peaks of 8-oxoG accumulation under conditions of hypoxia have been reported in active promoters linked to specific transcription factors [35, 36]. On a larger scale, studies found accumulation in GC and CpG island rich, early replicating DNA [38], but also in gene deserts and the nuclear periphery [39]. Some of these apparently contradicting conclusions may be explained through different levels of resolution, experimental systems, and methodology. So far, ionizing radiation-induced oxidative damage has not been addressed genome wide. In addition, base modifications, which have been processed into the more persistent AP-sites remain hidden from the previously used techniques.

To further our understanding of the molecular mechanisms underlying local mutation rate heterogeneity, direct and specific measurement of DNA damage types and repair intermediates is required at high resolution and on a genomic scale. Dissecting these mechanisms will help understand the local sensitivities of the genome and why certain regions appear to be protected.

## Results

### A genome-wide map of AP-sites

**Fig.1:**
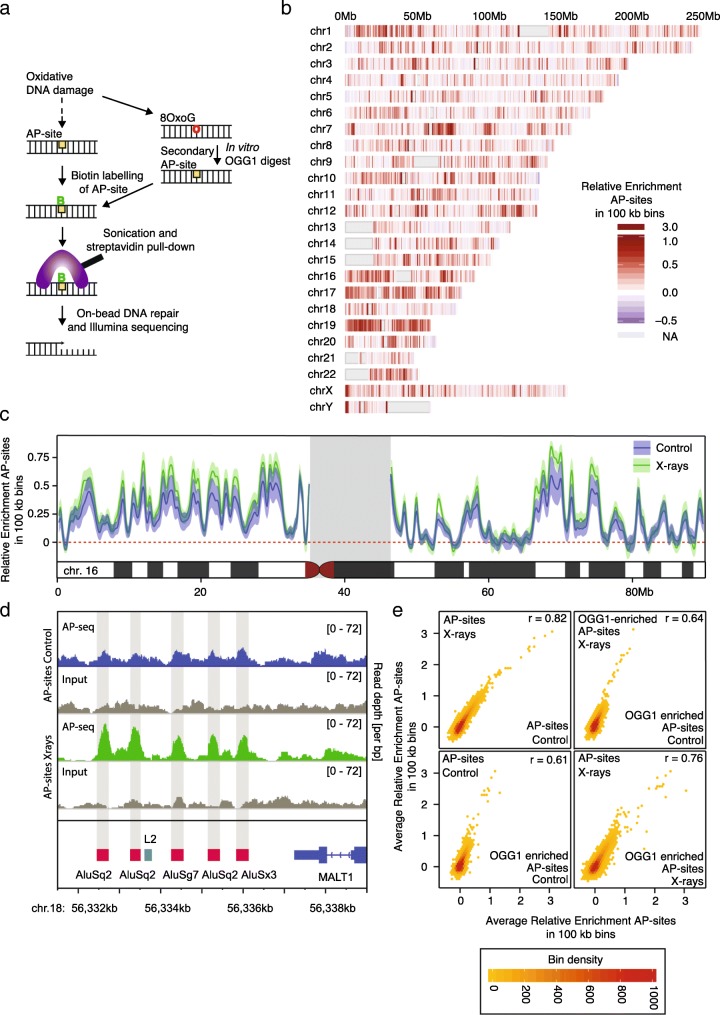
Oxidative damage is heterogeneously distributed at different scales of resolution. **a** Schematic of AP-seq, a new protocol to detect apurinic-sites (AP-sites). DNA containing these sites are biotin-tagged using an aldehyde reactive probe (ARP), fragmented, and pulled down with streptavidin. The enriched DNA is processed for sequencing and mapped to the reference genome. The damage level across the genome is quantified by assessing the number of mapped reads. To check for unprocessed 8-oxoG in addition to AP-sites, we perform an in vitro digest of extracted genomic DNA with OGG1 and repeat the AP-site pulldown. **b** Genome-wide map of AP-site distribution after X-ray treatment. The color scale represents the log2 fold change of normalized AP-seq enrichment over input (Relative Enrichment) in 100-kb bins across the human genome, averaged across biological replicates. Gray regions represent undefined sequences in the human genome, such as centromeres and telomeres. Damage levels are highly correlated between treatment conditions at 100-kb resolution. **c** More detailed view of AP-site distribution on Chromosome 16. Plot lines depict the average Relative Enrichment for AP-sites in samples after X-ray treatment (green) and without treatment (blue). Shaded boundaries show standard error of the mean for three biological replicates. Untreated and X-ray-treated samples display very similar damage profiles. **d** Genome browser views of damage distributions for untreated and X-ray-treated samples and their corresponding input samples across an 8-kb region upstream of MALT1. Damage levels are represented as unnormalized sequencing depth of the pooled biological replicates. At high resolution, it becomes apparent how sharp the damage levels rise over background at *Alu* elements after X-ray treatment, which leads to more distinct patterns than the broader distributed untreated control. **e** Scatterplots of the correlation in average Relative Enrichments of samples with differing treatment and OGG1-enrichment conditions. Damage levels are highly correlated across all conditions

To measure AP-sites across the genome, we developed an approach that specifically uses detection via a biotin-labelled aldehyde-reactive probe under pH neutral conditions, which has been well established for the specific detection of AP-sites since its development by Kubo et al. in 1992 [13, 42–46]; (Fig. 1a, Additional file 1: Figure S1, and Additional file 1: Figure S2A). While the same probe has been used to measure 5-formyl-cytosine (5-fC), the reactivity with 5-fC requires an acidic environment (pH5) with anisidine and 24-h incubation at 25 °C [47]. Under neutral conditions (pH7), 1 h at 37 °C, the probe is highly specific for the aldehydes occurring at AP-sites, which is the experimental condition we use (see Additional file 1: Figure S2 in Raiber et al. [47]); 5-fC is generated through the TET enzymes primarily in CpG islands and enhancers during early development, while the genome is demethylated [47, 48]. Under wildtype conditions, 5-fC levels do not exceed 20 ppm of cytosines [49]. Levels in adult tissues are much lower and anticorrelate with cell proliferation [50]. Due to the chemical specificity of the method and the expected absence of notable levels of 5-fC in the cell line used, 5-fC is not expected to contribute to measurements in the current study.

After fragmentation of genomic DNA, biotin-tagged DNA with the original damage sites was pulled down using streptavidin magnetic beads and prepared for high-throughput sequencing. The signal was quantified as the log2 fold change of normalized AP-site enrichment over input (Relative Enrichment), with positive values indicating regions of damage accumulation. As the distribution of damage was broad, showing only gradual changes beyond a number of hot spots in repetitive elements (see below and Figs. 1d and 3g, h), we analyzed the data using a binning approach and by assessing damage distribution relative to genomic features [51].

Figure 1b provides the first high-resolution, genome-wide view of AP-sites after X-ray treatment. Increase in damage levels has been confirmed using colorimetric measurements for AP-sites (Additional file 1: Figure S2B) and immunostaining for γH2AX foci (Additional file 1: Figure S2C). Measurements represent AP-sites acquired in response to X-ray treatment on top of background levels in HepG2 cells with good reproducibility (Additional file 1: Figure S3E). It immediately highlights the extreme variability in the relative density of AP-sites across the human genome: though the genome-wide mean Relative Enrichment is 0.1, local enrichments vary from less than − 0.6 to more than 3.0. Hot and cold spots are found across all chromosomes and do not appear to follow a particular distribution pattern: whereas chromosome 19 presents damage hot spots throughout the chromosome, on chromosome 7, we observe pericentromeric hot spots. Figure 1c shows a more detailed profile of chromosome 16, including distributions for treated and untreated samples. The profiles of the X-ray-treated samples indicate an overall treatment-dependent accumulation of damage; however, local relative distribution patterns of pre-existing background damage are maintained, suggesting that hot spots gain the most additional damage. In Fig. 1d, we zoom further into an 8-kb region upstream of the MALT1 gene. Here, differences between the treated and untreated samples become apparent, with damage after X-ray exposure particularly accumulating on *Alu* transposable elements in comparison to the surrounding sequence. Background AP-site levels indicate a similar albeit less pronounced trend of enrichment in *Alu* sequences. These plots exemplify how variable damage enrichments can be, with hot and cold spots ranging from ~ 50–500 bp to kilobase resolution.

To assess oxidative damage as the sum of AP-sites and 8-oxoG, we applied recombinant OGG1 in vitro to the extracted DNA (Fig. 1a). Under the conditions chosen, any remaining 8-oxoG is excised in a largely sequence-independent fashion after DNA extraction [52] to result in a set of secondary AP-sites and to a lesser extent the associated beta-elimination product [53]. In vitro, oligo-nucleotides with 8-oxoG-derived secondary AP-sites were pulled down with 12.1% recovery rate relative to input, an 11-fold increase as compared to the oligonucleotide containing guanine (Additional file 1: Figure S2A). This 1.1% background recovery rate represents for a large part heat-induced DNA damage, prompted by the oligonucleotide annealing step.

With the conversion of 8-oxoG into AP-sites, both damage types are measured simultaneously. However, any difference in enrichment patterns between the original and OGG1-enriched samples indicates the presence of unprocessed 8-oxoG in vivo. Although quantitatively different, the control and X-ray-treated samples are highly correlated overall (Fig. 1e). Moreover, the OGG1-enriched samples are very similar to the primary AP-sites, indicating that at 100-kb resolution, the OGG1 enrichment does not substantially alter the distribution. On these grounds, the AP-site measurements after X-ray treatment, the sample with the most pronounced patterns is shown as representative in the following analyses. OGG1-enriched samples are highlighted, where differences become apparent.

### Genomic features shape distribution of AP-sites and 8-oxoG

#### Damage accumulates preferentially in euchromatin but not heterochromatin

**Fig. 2:**
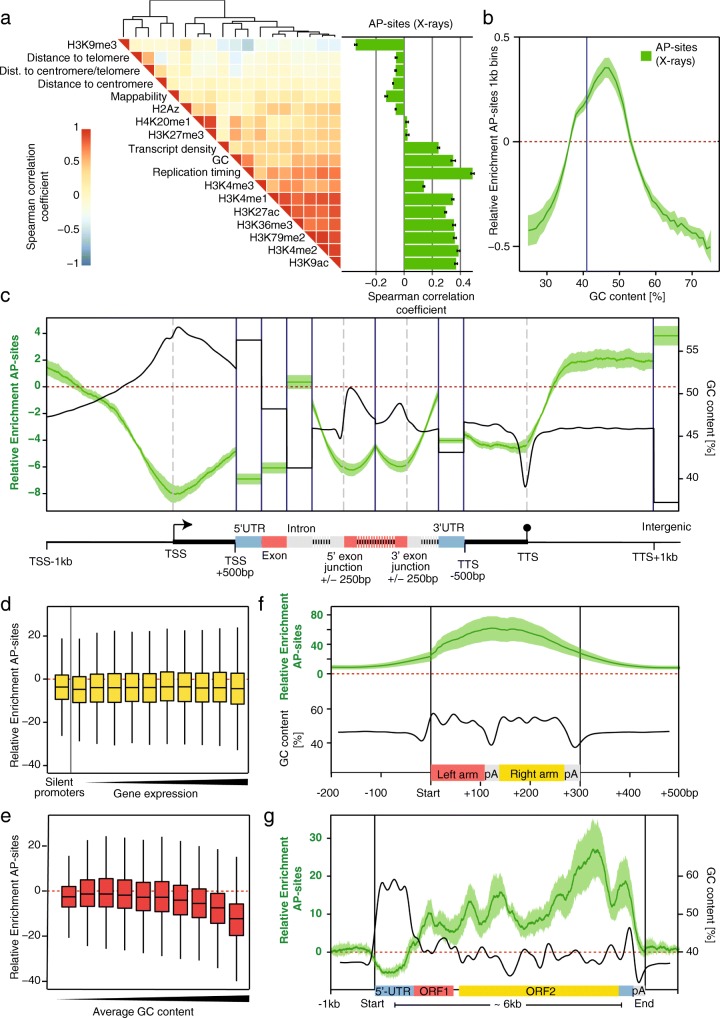
Oxidative damage distribution is associated with genomic features. **a** Bar plot displays the average correlation of damage levels with large-scale chromatin and other features in HepG2 cells at 100-kb resolution. Damage correlates with euchromatic features and anticorrelates with heterochromatic ones, the opposite of that observed for cancer SNVs. The heatmap shows the relationship between the features, grouped using hierarchical clustering. **b** The plot shows dependence between Relative Enrichment of damage and genomic GC content at 1-kb resolution. Damage levels increase with GC content and then surprisingly fall in high GC areas. The blue line marks the genomic average GC content of 41%. **c** Metaprofile of Relative Enrichment over ~ 23,000 protein-coding genes (*n*_genes_ = 23,056, *n*_promoters_ = 48,838, *n*_5UTRs_ = 58,073, *n*_exons_ = 214,919, *n*_introns_ = 182,010, *n*_3UTRs_ = 28,590, *n*_termination_ = 43,736, *n*_intergenic_ = 22,480). Damage levels for UTRs, exons, introns, and intergenic regions are averaged across each feature due to their variable sizes. GC content is depicted for the same regions smoothed with a Gaussian smooth ranging over 100 bp. Coding and regulatory regions are depleted for damage despite their increased GC content, whereas introns have near intergenic damage levels. **d**, **e** Boxplots depict damage levels at 48,838 promoters binned into unexpressed and expression deciles (**d**) and average GC content deciles (**e**). Promoters are defined as the transcriptional start sites ± 1 kb. Damage is not transcription-dependent but reduces with increasing promoter GC content. **f**, **g** Metaprofiles of Relative Enrichments and average GC contents across 848,350 *Alu* and 2533 *LINE* elements. There is a very large accumulation of damage inside these features. All panels display relative AP-site enrichment for X-ray-treated samples; for corresponding plots of the other treatment conditions, see Additional file 1: Figure S4A-D. Error bars and shaded borders show the standard error of mean across three biological replicates

To identify potential causes of variation across the genome, we compiled for the same HepG2 cell line a set of 18 genomic and epigenomic features previously associated with DNA damage, repair, and patterns of mutagenesis (Fig. 2a). Earlier studies reported that SNV density in cancer genomes was positively correlated with heterochromatin marks (e.g., H3K9me3) and negatively correlated with euchromatin marks (e.g., H3K4me3, H3K9ac) [29]. Here, AP-sites display the opposite trend, correlating with open chromatin and anticorrelating with closed chromatin, as previously suggested for 8-oxoG [38]. At first glance, it is surprising that SNVs and DNA damage should show opposing trends. However, mutagenesis is a multi-step process, with repair efficiency [54, 55] and replication accuracy [32] for instance being influenced by the chromatin state. Observations are upheld at higher resolutions for many features; for instance, Spearman’s correlation with H3K9me3 is −0.48 at 1-Mb resolution, −0.34 at 100-kb, −0.3 at 10-kb, and −0.14 at 1-kb resolution. For other features, these correlations break down; DNase I hypersensitivity correlates at low resolution (Spearman’s *r* = 0.5 and 0.3 at 1-Mb and 100-kb, respectively), but the relationship is lost at higher resolutions (*r* = 0.06 and −0.06 at 10-kb and 1-kb, respectively). This suggests that more detailed genomic features and functional elements also play a role in shaping the local damage distributions.

#### Damage enrichment is GC content dependent

As oxidative damage predominantly occurs on guanines [1], base content is expected to be a prime determinant of genome-wide distribution. The heatmap in Fig. 2a shows that this is true in general, with average damage levels in 100-kb windows correlating with GC content (Spearman’s *r* = 0.37). However, closer examination shows a more complex relationship: in Fig. 2b, we plot average damage levels in 1-kb windows against their GC content. While there is a clear increase in damage as GC content rises from 25 to 47%, this relation breaks down above 47% GC and damage levels drop sharply. This indicates that while there is a larger proportion of the receptive base with increasing GC content, damage in regions of high GC content cannot be explained by base composition alone.

#### Gene promoters and bodies show selective protection from damage

Next, we interrogated damage distributions over coding regions by compiling a metaprofile for 23,056 protein-coding genes (Fig. 2c and Additional file 1: Figure S4B). The analysis reveals rigid compartmentalization, with relative damage levels varying substantially between elements and opposed to GC content distribution. Damage is dramatically reduced within genes compared to flanking intergenic regions (Relative Enrichment = 3.8), most prominently at the transcriptional start (Relative Enrichment = − 8.0), 5′ UTRs (Relative Enrichment = − 6.9), exons (Relative Enrichment = − 6.1), and termination sites (Relative Enrichment = − 5.8). In stark contrast, introns show high damage (Relative Enrichment = 0.4), though still below intergenic levels. Intron-exon junctions are accompanied by steep transitions in damage indicating the sharp distinction between coding, regulatory, and non-coding regions (Relative Enrichment changes from −6.0 to −0.5 within 300 bp around the 3′-exon junction). Damage levels rapidly rise again downstream of termination sites towards intergenic regions (Relative Enrichment shifts from − 4.3 to 2.0 within 500 bp).

Promoters and transcription start sites have the lowest damage levels of any functional element in the genome (average Relative Enrichment = − 8.0 compared with intergenic average of 3.8), similar to what has been shown for 8-oxoG and alkylation adducts together with their resulting AP-sites in yeast [37, 55]. Unlike SNVs and other damage types, which decrease with rising gene expression levels, we do not detect an association between AP-sites and expression (Fig. 2d and Additional file 1: Figure S5A). There is a substantial GC content effect (Fig. 2e and Additional file 1: Figure S5B), but in contrast to expectations from base composition alone, damage levels fall as GC content rises (Relative Enrichment = 1.1 at 45% GC and Relative Enrichment = − 12.6 at > 64% GC).

#### Retrotransposons accumulate large amounts of damage

Retrotransposons [56] provide a fascinating contrast to coding genes: long interspersed nuclear elements (*LINEs*) possess similar structures to genes with an RNA Pol II-dependent promoter and two open reading frames (ORFs), whereas short interspersed nuclear elements (*SINEs*) resemble exons in their nucleotide compositions and presence of cryptic splice sites. Unlike coding genes though, *LINEs* and *SINEs* accumulate staggeringly high levels of damage. *Alu* elements, the largest family among *SINEs*, show by far the highest damage levels of any annotated genomic feature: a metaprofile of > 800,000 *Alu* elements in Fig. 2f (and Additional file 1: Figure S4C) peaks at an average Relative Enrichment of 59, much higher than the genomic average of 0.1. The damage profile rises and falls within 500 bp. Interestingly, unlike promoters and exons, enrichment in intronic *Alus* increases with GC content (Additional file 1: Figure S5C). Similar to *Alus*, a metaprofile of > 2500 *LINE* elements in Fig. 2g and Additional file 1: Figure S4D displays heterogeneous but high levels of damage accumulation: like coding genes, there is reduced damage at promoters (average minimum Relative Enrichment = − 5.2), but in contrast to genes, there is a gradual increase in damage from the 5′ to 3′end, peaking at a Relative Enrichment of 26.9 near to the end of the second ORF. A difference in the distribution pattern between AP-sites and OGG1-enriched AP-sites suggests differential patterns of 8-oxoG accumulation, possibly through formation of secondary DNA structures (see below) in *LINE* elements [57].

Retrotransposons, though usually silenced through epigenetic mechanisms [58], can be activated through loss of repair pathways [59], by DNA damage in general [60] and ionizing radiation in particular [61]. How DNA damage or repair affects such silencing mechanisms is currently unknown. One might speculate that DNA damage at these positions could lead to unwanted *LINE* transcription, for instance through repair-associated opening of chromatin. These distinct and unique damage patterns of both protection and strong accumulation of damage within one functional element suggest the existence of targeted repair or protective mechanisms that are unique to retrotransposons.

#### Transcription factor binding sites, G-quadruplexes, and other regulatory sites

**Fig. 3:**
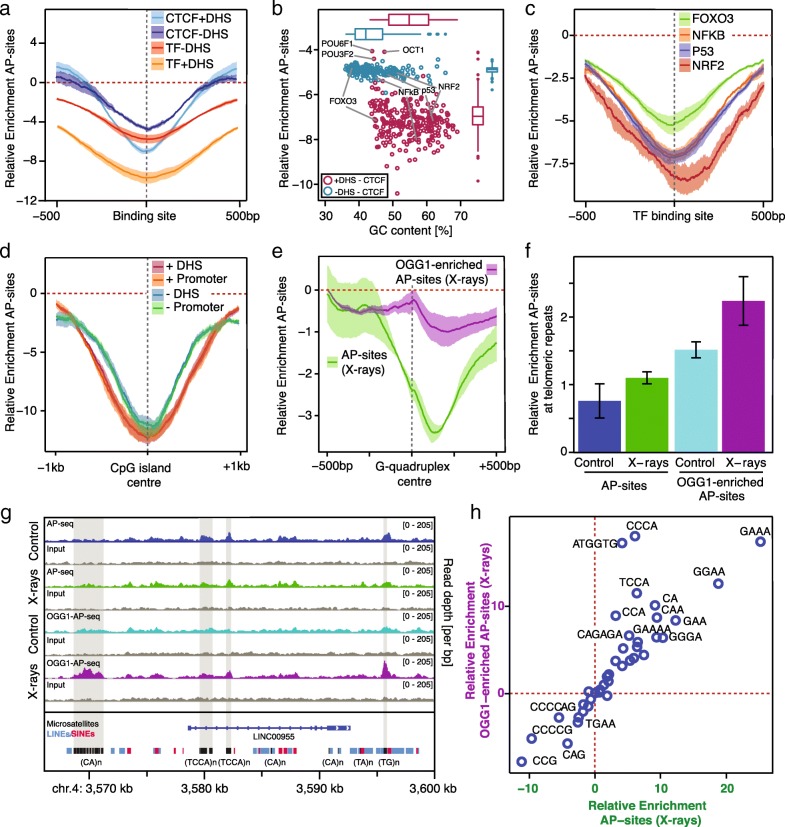
Oxidative damage distribution is associated with regulatory sites and repeats. **a** Metaprofiles of Relative Enrichments centered on CTCF and DNA binding sites within and outside DNase hypersensitive regions (DHS; *n*_CTCFinDHS_ = 37,763, *n*_CTCFnotDHS_ = 10,908, *n*_TFbsInDHS_ = 253,613, *n*_TFbsNotDHS_ = 5,463,612). Damage levels are reduced around binding sites. Shaded borders show the standard error of mean across biological replicates. **b** Scatter plot of average Relative Enrichments and GC contents ± 500 bp of binding sites for each transcription factor excluding those within 500 bp of a CTCF binding site as these represent a special case (see Additional file 1: Figure S5D). Binding sites are separated into within and outside DNase hypersensitive sites. Damage levels are universally reduced regardless of transcription factor, with particularly lowered levels for actively used sites in DHS regions. **c** Metaprofiles centered on binding sites for four selected transcription factors. **d** Metaprofiles centered on CpG islands, within and outside promoters and DHS regions (*n*_DHS_ = 17,565, *n*_NotDHS_ = 9878, *n*_Promoter_ = 14850, *n*_NotPromoter_ = 12,593). Damage levels are reduced regardless of location and accessibility. **e** Metaprofiles centered on predicted G-quadruplexes (*n* = 359,449). There are asymmetrically reduced damage levels for AP-sites, but not for OGG1-enriched AP-sites. **f** Bar plots of average Relative Enrichments in G-quadruplexes at telomeric repeats across the four treatment and processing conditions. Damage levels are increased in OGG1-enriched samples. Error bars show the standard error of mean across three biological replicates. **g** Genome browser views of unnormalized damage levels in ~ 30-kb locus surrounding LINC00955, including microsatellite repeats. Some groups of microsatellites accumulate large amounts of damage and reduced 8-oxoG processing. **h** Scatter plot displaying average damage levels in different microsatellite types for the AP-site and OGG1-enriched samples. Reverse complementary repeats were assigned to the alphabetically first repeat. Most types display similar damage levels in the two processing conditions; however, several display elevated damage in the OGG1-enriched sample. All panels display measurements for X-ray-treated samples, unless indicated otherwise. For corresponding plots of CpG islands in general and G-quadruplexes with the other treatment conditions, see Additional file 1: Figure S4E and F

Next, we examine the most detailed genomic features previously associated with mutation rate. In Fig. 3a–c and Additional file 1: Figure S5D, we assess the impact of DNA-binding proteins: there is a universal U-shaped depletion of damage levels ± 500 bp over the binding site regardless of the protein involved, suggesting that the act of DNA binding itself is a major protective factor. We find the greatest reduction in damage for actively used binding sites that overlap with DNase hypersensitive regions in the HepG2 cell line. However, a smaller reduction is also present for inactive sites, indicating that the effects go beyond simple DNA binding. It is notable that the accessibility of the site overrides the contribution of the GC content to damage levels (Fig. 3b).

GC-rich features are particularly interesting because of the complex relationship between GC content, protein binding, and damage levels. CpG islands are frequently located in promoters and display reduced damage (Fig. 3d and Additional file 1: Figure S4E). Most surprising is the dramatic reduction in damage at CpG islands outside promoters and DNase-hypersensitive regions, indicating that the localization in promoters is not the main reason for damage reduction; in fact, it is possible that the reduction in damage for high-GC promoters might be explained by the presence of CpG islands and not vice versa.

Another feature of GC-rich sequences are G-quadruplexes (G4 structures) formed by repeated oligo-G stretches. G-quadruplexes are prevalent in promoters [62], *LINE* retrotransposons [57], and telomeric regions [63], where they impact telomere replication and maintenance [64]. A metaprofile for > 350,000 predicted G4 structures display an asymmetric reduction in damage, in which the minimum occurs just downstream of the G-quadruplex center (Fig. 3e and Additional file 1: Figure S4F). In line with hypoxia-induced 8-oxoG accumulation at G4 structures [35], we identify G-quadruplexes as one of the few features with clear differences between the 8-oxoG and AP-site distributions, exhibiting a particular enrichment at the center of G4 structures. This finding is particularly relevant for telomeric repeats (Fig. 3f), where oxidized bases impact on telomerase activity and telomere length maintenance [65]. These repeats are thought to form G4 structures, but in contrast to quadruplexes in general, telomeres present with a mild increase in AP-sites after X-ray treatment (average Relative Enrichment = 1.1) and stronger enrichment of OGG1-enriched AP-sites (average Relative Enrichment = 2.3).

Microsatellites are 3–6-bp sequences that are typically consecutively repeated 5–50 times. Whereas GC-rich microsatellite repeats show generally reduced damage, most simple repeats show an accumulation of damage; this is depicted for individual repeat sites at the *LINC00955* locus (Fig. 3g). The motifs (GAA)_n_, (GGAA)_n_, and (GAAA)_n_ accumulate the largest amounts of damage (Fig. 3h). Interestingly, specific sequences display preferential damage enrichment in the OGG1-enriched samples, such as (CCCA)_n_ and (ATGGTG)_n_. Microsatellites are capable of forming non-B-DNA structures such as hairpins [66]; we suggest that changes in the DNA’s local structural properties impair 8-oxoG processing on these genomic features with possible regulatory functionality.

#### Chromatin architecture

**Fig. 4:**
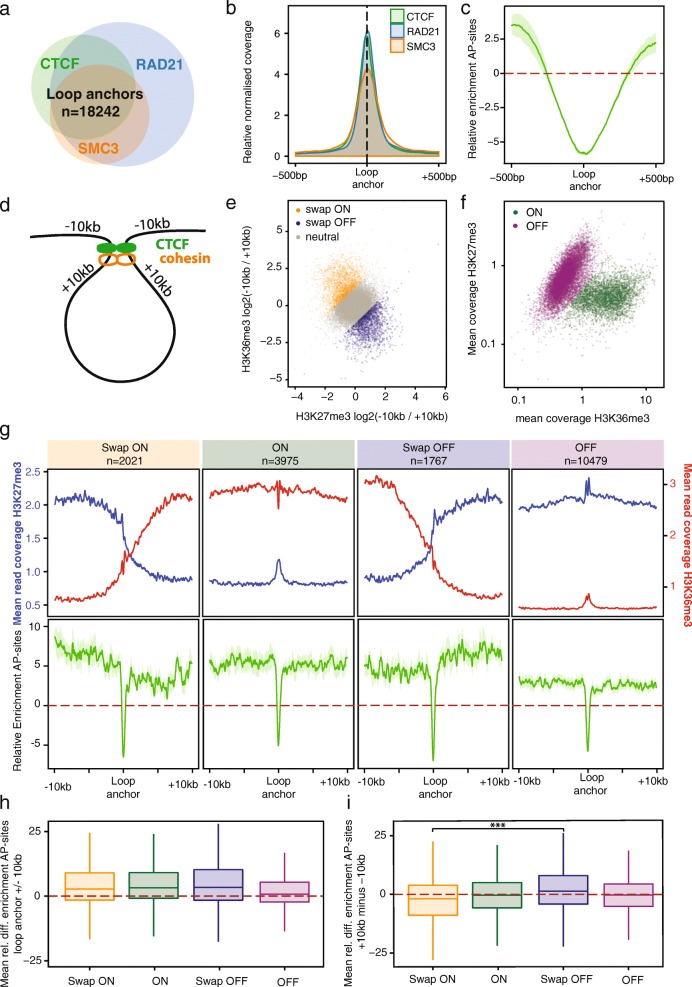
Oxidative damage patterns follow chromatin changes at chromatin loop anchors. **a** Loop anchors are defined by overlaps of a canonical CTCF motif with CTCF peaks as well as the cohesin components RAD21 and SMC3. Loop anchor sites (*n* = 18,242) were localized to the center of the CTCF motif and oriented accordingly. **b** Mean read coverage around the loop anchors is depicted for all three components. **c** AP-site distribution, determined as Relative Enrichment of AP-sites after X-ray treatment. For corresponding plots depicting the other treatment conditions, see Additional file 1: Figure S4G. **d** Based on the orientation of the loop anchor, chromatin status was determined outside (− 10 kb) and inside (+ 10 kb) of the chromatin loop. **e** As markers of active and inactive chromatin, the log2 ratios of H3K36me3 and H3K27me3 read coverage outside and inside the loop are depicted relative to the loop anchors. Their ratio is taken as a cut-off to categorize the insulation properties of the loop anchor. Loop anchors with a differential log2 ratio of 1.2 are defined as anchors that lead to a swap from inactive to active chromatin “swap ON” (*n* = 2021). A differential log2 ratio below − 1.2 is separating anchors that lead to a swap from active to inactive chromatin “swap OFF” (*n* = 1767). Neutral loop anchors were differentiated further as depicted in **f**. Neutral loop anchors that do not lead to a change in chromatin are differentiated by their mean H3K36me3 and H3K27me3 coverage ± 10 kb. Loops are defined to be in inactive chromatin “OFF” (*n* = 10,479), if log2(H3K27me3/H3K36me3) exceeds 2. Otherwise, loop anchors are considered to be in open chromatin “ON” (*n* = 3975). **g** H3K27me3 and H3K36me3 mean coverage distribution over the loop anchor classification illustrates the changes of chromatin states. Comparison to AP-sites, determined as relative enrichment after X-ray treatment (mean ± standard error of the mean), shows a reduction of AP-sites at a change into active chromatin. Loop anchors in inactive chromatin are low in AP-sites, despite inactive chromatin adjacent to active chromatin showing the highest damage levels. AP-sites are quantified in **h** as mean relative enrichment at the loop anchors ± 10 kb, and changes in AP-site prevalence are quantified in **i** as the mean relative differential enrichment at loop anchor + 10 kb minus loop anchor − 10 kb with significantly different changes of damage levels between the “swap ON” and “swap OFF” categories, *p* < 0.001 by Wilcoxon rank test, indicated by asterisks

Chromatin loop anchors represent a special feature in DNA repair. On the one hand, tight binding by the cohesin complex is described to block nucleotide excision repair [67]; on the other hand, DNA damage response and repair organization were shown to originate from loop anchors [68]. Investigating the effect of chromatin organization on AP-site distribution, we used overlapping peaks of CTCF, RAD21, and SMC3 as a proxy for the location of 18,242 chromatin loop anchors (Fig. 4a, b). We found damage strongly reduced at the loop anchors themselves (Relative Enrichment less than − 5; Fig. 4c) with a steep increase to a Relative Enrichment of ~ 2.5 within 500 bp. Stratifying loop anchors by the chromatin states on both sides based on H3K36me3 and H3K27me3 coverage within 10-kb of the anchor (Fig. 4d–f) confirms the previous findings of increased AP-sites in active chromatin (Fig. 4g–i). However, in chromatin loops that insulate active from inactive chromatin, AP-site distribution reduces with chromatin activity, irrespective of whether the inside or outside of the loop represents the active component. It is therefore conceivable that beyond the protection of the loop anchor itself, protection from or repair of AP-sites might be given preference in the active chromatin compartment.

### SNVs in oxidative damage-dependent cancers reflect underlying damage profiles

Lastly, we address how the distribution of oxidative DNA damage is reflected in the landscape of SNVs in cancer genomic data. We compiled a dataset of 8.6 million C-to-A transversions, the major mutation type caused by oxidative damage [69], from 2401 cancer genomes [70]. These were stratified by the proportion attributable to COSMIC Mutational Signature 18 [71, 72], which has been suggested to arise from genomic 8-oxoG mispairing with adenine [73, 74].

**Fig. 5:**
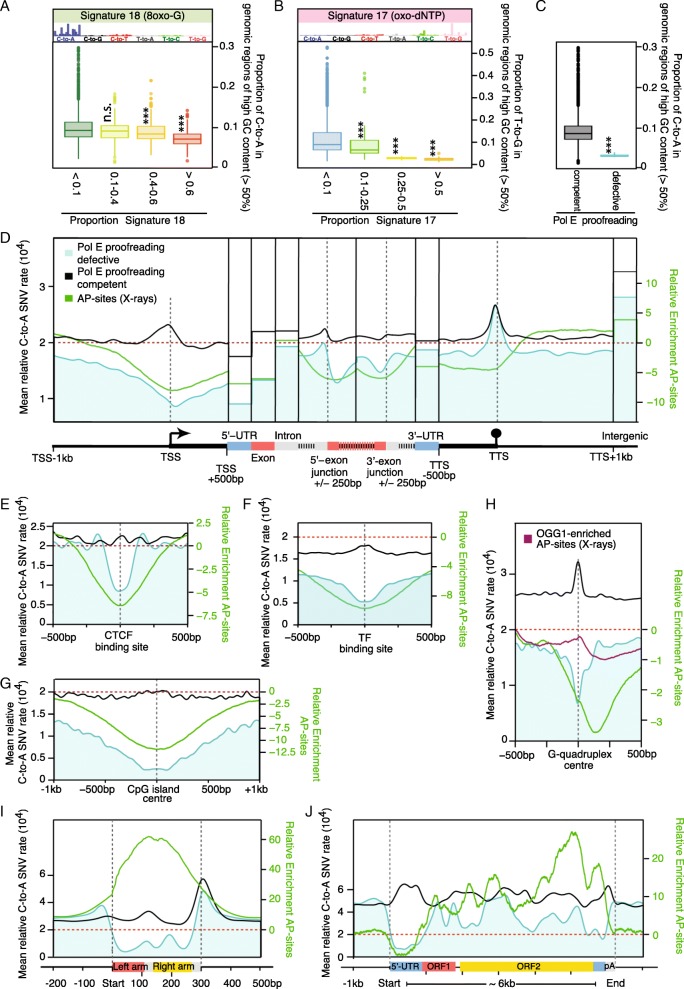
Oxidative damage patterns are reflected in cancer mutagenesis. **a** Boxplots of the proportion of C-to-A SNVs (including the reverse complement G-to-T) in genomic regions of high GC content (> 50%). Tumor samples are separated into four groups according to Mutational Signature 18 contributions (*n*_< 0.1_ = 1398, *n*_0.1–0.4_ = 322, *n*_0.4–0.6_ = 540, *n*_> 0.6_ = 141). Asterisks indicate significance of *p* < 0.001 by Wilcoxon rank test comparing the different Signature 18 proportions to Signature 18 < 0.1. Bar plots depict the original COSMIC mutational signatures. Tumors that are high in Signature 18 display lower proportions of SNVs in GC-rich regions, while tumors with mutations in OGG1, APEX1, or FEN1 show higher proportions. **b** Boxplots of the proportion of T-to-G SNVs (including the reverse complement A-to-C) in genomic regions of high GC content (> 50%). Tumor samples are separated into four groups according to Mutational Signature 17 contributions (*n*_< 0.1_ = 2255, *n*_0.1–0.25_ = 78, *n*_0.25–0.5_ = 59, *n*_> 0.5_ = 9). Asterisks indicate significance of *p* < 0.001 by Wilcoxon rank test comparing the different Signature 17 proportions to Signature 17 < 0.1. Tumors that are high in oxidative damage signatures display lower proportions of their respective signature mutations C-to-A or T-to-G in GC-rich regions. **c** Boxplots of the proportion of C-to-A SNVs in genomic regions of high GC content (> 50%). Tumor samples are separated into those that are Pol E-proofreading defective (*n* = 8) and to all other tumors (*n* = 2694). Asterisks indicate significance of *p* < 0.001 by Wilcoxon rank test comparing the PolE proofreading deficient to competent. Tumors that are proofreading defective and high in Signature 18 display lower proportions of SNVs in GC-rich regions. **d** Metaprofile of SNV rates over ~ 23,000 protein-coding genes in proofreading-defective and control tumors. The damage profile is overlaid for comparison. The oxidative damage-dependent SNV profiles in proofreading-defective tumors show similar distributions to AP-sites, whereas the pattern is lost in control tumors. **e**–**h** Metaprofiles of SNV rates centered on CTCF binding sites (*n* = 48,671; **e**), transcription factor-binding sites in DHS regions (*n* = 253,613; **f**), CpG islands (*n* = 27,443; **g**), and G-quadruplex structures (*n* = 359,449; **h**). SNV profiles in proofreading-defective tumors mimic the damage profiles. **i**, **j** Metaprofiles across 848,350 *Alu* (**i**) and 2,533 *LINE* elements (**j**). SNV rates in proofreading-defective tumors are reduced compared with damage profiles

In most tumors, about 9% of C-to-A SNVs occur in regions of high GC content (Fig. 5a). However, tumors display decreasing proportions of SNVs in GC-rich regions with rising amounts of Signature 18 exposure (Fig. 5a), following the expected trend for oxidative damage.

In addition, we investigated 4.8 million T-to-G transversions and related their GC content preference to Signature 17 (Fig. 5b). This signature has been associated with oxidative DNA damage related to oxidative stress induced by gastroesophageal reflux [75, 76]. Signature 17 is believed to arise from incorporation of modified bases from an oxidized dNTP pool during replication. Hoogsteen base pair-derived mismatches between 8-oxo-dGTP and adenine that evade repair can result T-to-G mutations. For all tumors, a median proportion of 9% of T-to-G mutations occur in GC-rich DNA. Whilst Signature 17 however contributes more than a quarter of all T-to-G mutations, this median falls below 3%, more than twice the decline expected from sequence content alone (Additional file 1: Figure S9F). In conclusion, mutations from both signatures linked to oxidative DNA damage are depleted in GC-rich DNA, resembling the observed AP-site distribution. Interestingly, the impact of Signature 17 is dependent on damaged nucleotide incorporation and repair efficiency. It is not dependent on oxidative damage impact on genomic DNA. Therefore, this analysis indicates GC content preferences of oxidative damage repair.

Lastly, we compiled a dataset of 3.4 million C-to-A transversions from eight cancer genomes defective in polymerase epsilon (Pol E) activity. Under normal conditions, Pol E-proofreading prevents 8-oxoG-A mismatches, but in the absence of this activity, such mismatches are expected to result in C-to-A mutations of yet unknown proportion [71]. Thus, we investigated whether the distribution of SNVs in the absence of Pol E-proofreading would follow the underlying oxidative damage pattern and reflect the local differences in damage susceptibility or repair preferences [72].

In most tumors, about 9% of C-to-A SNVs occur in regions of high GC content (Fig. 5c; however, the proportion drops to just 3% among Pol E-defective tumors, in line with the unexpected depletion of oxidative damage in these genomic regions (Fig. 2b). We also observed that damage is preferentially distributed in euchromatin at 100-kb resolution, whereas SNVs tend to accumulate in late replicating heterochromatin; unsurprisingly at this resolution, the damage and SNV densities are anticorrelated (Spearman’s *r* = − 0.49 and − 0.45 for proofreading-defective and control tumors, respectively). Reduced mutation rates in high GC content DNA do however occur irrespective of replication timing (Additional file 1: Figure S8).

We focused on the proofreading-defective and control tumor samples for the high-resolution genomic features, as they contain the largest numbers of SNVs. We also related these patterns to the equally prominent C-to-T mutations (Additional file 1: Figure S7), which are thought to arise from different mechanisms, e.g., uracil bypass and true C-dA misincorporation [77, 78], mechanisms that are partially dependent on base excision repair. In protein-coding genes, the SNV distribution for Pol E-defective tumors is remarkably similar to the damage profiles (Fig. 5d and Additional file 1: Figure S7A): decreased rates of C-to-A transversions at the TSS, 5′-UTR, and exons and increased rates in introns. The profile is lost in control tumors: we speculate that bulky adducts or strand breaks—a distinct form of damage—cause the accumulation of SNVs at the promoter. Interestingly C-to-T SNVs show opposite trends in exons (Additional file 1: Figure S7A). C-to-A SNVs are also depleted from GC-rich genomic features in Pol E-defective tumors, including CTCF binding sites, transcription factor binding sites, CpG islands, and G-quadruplexes. The patterns are lost in the controls (Fig. 4e–h and Additional file 1: Figure S7B-E). The difference between the two tumor sets indicates that at high resolution, the distribution of distinct damage types dominates the ultimate SNV profiles. However, there is a striking divergence from damage distributions in retrotransposons (Fig. 5i, j and Additional file 1: Figure S7F and G); whereas above we observed high levels of damage in *Alus* and *LINEs*, there appears to be increased safekeeping, leading to lower levels of C-to-A mutations. This pattern is lost in the control tumors.

## Discussion

Our results demonstrate the feasibility of measuring AP-sites as a marker of oxidative damage and its first repair intermediate across a genome at ~ 250-bp resolution and high specificity. Damage is strongly reduced in regions of high GC content, which also depends on DNA accessibility. Previous measurements of oxidative damage using antibodies for 8-oxoG agree with the accumulation of oxidative damage in open, early replicating DNA [38]. Other studies describe oxidative damage accumulation in the nuclear periphery and gene deserts [39] as well as in certain promoters [35, 36]. Addressing the more persistent AP-sites, we find open DNA increasingly damaged at the 100-kb scale. However, unprocessed 8-oxoG accumulates at potential DNA secondary structures, such as G-quadruplexes, telomeres, and in certain simple repeats. The promoters identified to accumulate 8-oxoG using candidate gene approaches, and peak calling [35, 36] largely contains such predicted secondary structures, e.g., the mouse VEGF promoter, which Pastukh et al. characterized during hypoxia [35], showing regulation through a mechanism involving 8-oxoG accumulation at its G4 structure [79]. Apart from these exceptional genes, there is no evidence that promoters in general show 8-oxoG accumulation. On the contrary, in yeast, Wu et al. [37] showed generally reduced 8-oxoG levels in promoters. For AP-sites, we describe a GC content-dependent reduction of oxidative damage levels, a pattern that does not change upon additional OGG1 treatment. Similar profiles for methyl methane sulfonate (MMS) induced methyl adducts, and their resulting AP-sites in yeast suggest a mechanistic basis in increased base excision repair activity at promoters [55]. Consequently, promoters and other high GC content DNA are likely reduced in AP-sites rather through a mechanism of increased repair activity than protection from damage impact. Protection of such region from Signature 17-derived mutations also supports a mechanistic interpretation that focuses on DNA repair preferences.

Exons also showed a striking protection from AP-sites, with a strong contrast to introns. This protection is equally not transcription, but GC content dependent. One might speculate that the GC content itself may be involved in either protecting the relevant genomic regions or directing repair. The difference between exons and *Alu* retrotransposons is therefore of particular interest. Although equal in size and GC content, they display distinctly different AP-site patterns, exons being protected and *Alus* accumulating large amounts of damage. Therefore, the biochemical determinant differentiating between exons and *Alus* is likely to be found in epigenetic mechanisms, such as exon-associated histone marks, e.g., H3K36me3 or through direct interaction with RNA processing or splicing, as it is increasingly suggested for several mechanisms of DNA repair [72, 80].

In addition to the considerable feature-dependent variability in damage rates, we are able to relate them directly to patterns of SNV occurrences in cancer genomes. At the 100-kb scale, euchromatin has increased damage levels, yet fewer SNVs. Euchromatic DNA is known to be replicated more accurately due to increased postreplicative mismatch repair [81, 82]. In addition, one could speculate that exposure to oxygen radicals, but also better accessibility for repair machinery, may lead to this discrepancy [81]. At the 10-kb to 300-bp resolution, we find reduced damage levels in functional elements such as coding sequences, promoters, and transcription factor binding sites. The heterogeneity likely results from changes in the balance of damage susceptibility and repair rates at different genomic regions.

Locus-specific oxidative damage is distinct from damage types repaired by other pathways such as nucleotide excision repair (NER). For instance, AP-site levels are seemingly independent of gene expression, whereas nucleotide excision repair can be coupled to transcription [83]. Moreover, for NER, Sabarinathan and Perera reported UV-dependent mutation hot spots around transcription factor binding sites explained by hindered access of the repair machinery. For AP-sites, we observe the opposite: protection of the same regions. Such hot spots are probably prevented through partial inaccessibility of the DNA to oxygen radicals, which is not the case for UV light. Alternatively, increased repair activity in these regions may lead to a reduction of oxidative damage levels. The mechanistic basis of how genomic features are identified for protection still remains elusive. We find that a complex interaction of sequence content, DNA accessibility, protein binding, exon recognition, and chromatin architecture modulates protective effects.

Intriguingly, large amounts of damage accumulate in *LINEs* and *Alus*. DNA damage accumulation at these sites would suggest not only effects on mutagenesis, but also on silencing of these transposable elements [59–61]. Notably, an epigenetic function in hypoxia-induced gene expression at G4 structures has been suggested for 8-oxoG [35, 79]. At these sites and other potential non-B-DNA structures, we detected elevated signals in the OGG1-enriched samples, confirming the in vivo accumulation of 8-oxoG [35] and suggesting that 8-oxoG-processing is impaired. It is interesting to speculate that these sites may have acquired a regulatory function beyond accumulating mutations.

In conclusion, we have established a robust method to measure AP-sites, and indirectly 8-oxoG together with AP-sites, in a genome-wide manner. With minor modifications, it will be suitable for detecting any base modification that can be excised with a specific glycohydrolase. Identifying the pathways that lead to selective repair fidelity and protection of functional elements will not only provide insights into basic mutagenesis but will also allow us to identify any regulatory characteristics of 8-oxoG and AP-sites as epigenetic marks.

## Methods

### Cell culture and X-ray treatment

HepG2 cells were chosen for these experiments on the basis of the availability of additional data from the ENCODE project. In addition, HepG2 cells are preferentially used for DNA damaging compounds that require enzymatic activation (e.g. aflatoxin), which may allow comparison of pathways and damage types in later studies.

HepG2 cells were cultivated at 37 °C and 5% CO2 in Dulbecco’s modified Eagle medium (DMEM; Invitrogen) supplemented with 1% essential amino acids, 1% pyruvate, 2% penicillin/streptavidin, and 10% heat-inactivated fetal bovine serum (FBS). Approximately 1 × 10^6^ cells were exposed to 6 Gy X-ray using a SOFTEX M-150WE in triplicates. Triplicate samples of untreated control cells were processed in parallel, excluding irradiation. Cells were harvested 30 min post-treatment.

Successful treatment was confirmed using immunocytochemical staining for γH2AX. Cells were fixed in 2% formalin in phosphate-buffered saline pH 7.2 (PBS). Blocking and permeabilization were performed with 0.2% fish skin gelatin, 0.5% bovine serum albumin (BSA), and 0.5% Triton X-100 in PBS. Staining for γH2AX was done with a mouse monoclonal antibody (Millipore #05 636) in 1:2000 dilution and stained with a FITC-coupled secondary antibody. Nuclear staining with DAPI was included in the mounting medium (ProLong Gold Antifade Mountant, ThermoFisher, catalog number P36931). Images were taken with an Olympus FV1000 microscope.

### In vitro pulldown of damaged oligonucleotides

Oligonucleotides with defined damage sites were used to determine the efficiency of the pulldown in vitro. The sequence was adapted from the 59mer used by Guibourt et al. [35] with additional M13 primer binding sites (Additional file 2: Table S1).

Oligonucleotides were hybridized at a concentration of 50 μM for 2 min at 94 °C and gradually cooled to room temperature. Ten picomoles of the double-stranded 8-oxoG-containing oligonucleotide was enzymatically digested with one unit recombinant OGG1 (New England Biolabs, catalog number M0241L) in New England Biolabs (NEB)-buffer 2 and bovine serum albumin (BSA) and simultaneously tagged with biotin using 5 mM Aldehyde Reactive Probe [42] (ARP; Life Technologies, catalog number A10550) for 2 h at 37 °C. The control oligonucleotide with guanine was tagged with biotin using 5 mM ARP in TE-buffer containing 10 mM Tris and 1 mM EDTA, pH8. Samples were purified using a ChargeSwitch PCR Clean-up Kit (Invitrogen, catalog number CS12000).

Half of the sample (up to 5 pmol) was saved as input. The other half was processed for pulldown using 5 μl MyOne Dynabeads (Life Technologies, catalog number 65601). Beads were washed three times with 1 M NaCl in TE-buffer and re-suspended in 2 M NaCl in TE-buffer and then added to the equal volume of oligonucleotide solution. The pulldown was performed for 10 h at room temperature. The beads were washed three times with 1 M NaCl in TE-buffer. To release the DNA from the beads, the beads were incubated in 95% formamide and 10 mM EDTA for 10 min at 65 °C and subsequently purified using the ChargeSwitch PCR Clean-up Kit. Two percent of the pulldown was used as template for qPCR. qPCR was performed in 25-μl reactions using a Biorad CFX96 Real-Time System with 2× Maxima SYBR Mastermix (ThermoFisher, K0221) and 0.3 μM primers. Of the saved input, 1% was used as template for qPCR.

Recovery of input was calculated as 2^−ΔCT^ with the differential between pulldown and input. The data were subsequently normalized to the guanine-oligonucleotide as it represents the background pulldown efficiency including background from spontaneous AP-sites that presumably arise as a result of the heating step used to anneal the oligonucleotides.

### AP-site colorimetric measurement and AP-Seq

Total genomic DNA was extracted using a Blood and Tissue Kit (Qiagen, catalog number 69506), and genomic DNA was kept on ice during the process. Antioxidants were not applied in this experiment to avoid artifacts through sequence-specific effects. Since treated samples and the untreated control are exposed to the same technical artifacts from sample processing, these should be accounted for in the data analysis. 5.7 μg of genomic DNA was tagged with biotin using 5 mM Aldehyde Reactive Probe [42] (ARP; Life Technologies, catalog number A10550) in phosphate-buffered saline (PBS) for 2 h at 37 °C. Genomic DNA was then purified using AMPure beads (Agencourt, catalog number A63882) with 1.8× bead solution and 2× 70% ethanol washing; beads were not allowed to dry to prevent DNA from sticking.

Colorimetric measurement of AP-sites was performed using a commercial kit (abcam, catalog number ab65353) following the manufacturer’s protocol starting from the DNA binding step with 60 μl and 0.1 μg/ml. Optical density at 650 nm was normalized using the standard curve of defined damage sites. From the resulting values, the log2 fold difference to the control mean was calculated and depicted as mean and standard error of the mean. These data were not used for normalization purposes of the sequencing experiments due to the general semi-quantitative nature of this method.

For AP-Seq, biotinylated DNA was fractionated using a Covaris fractionator in 130 μl for a mean fragment length of 300 bp. After separating 30 μl for sequencing as the input sample, the remaining DNA was used for biotin-streptavidin pulldown, using MyOne Dynabeads (Life Technologies, catalog number 65601). One hundred twenty microliters of beads (10 μl per sample) were washed three times with 1 ml 1 M NaCl in Tris-EDTA buffer (TE-buffer) and re-suspended in 100 μl 2 M NaCl in TE and then added to 100 μl of the sonicated DNA. Samples were rotated at room temperature for 10 h. Subsequently, the beads were washed three times with 1 M NaCl in TE and finally re-suspended in 50 μl TE for library preparation.

For the in vitro OGG1-enrichment (OGG1-AP-Seq), 10 μg of genomic DNA was digested with recombinant OGG1 (New England Biolabs, catalog number M0241L). 0.1 μg enzyme was taken for 1 μg of genomic DNA in New England Biolabs (NEB)-buffer 2 and bovine serum albumin (BSA) for 1 h, 37 °C. Such conditions for the enzymatic digest should account for sequence content-dependent differences in enzyme activity as described by Sassa et al. [52]. Digested DNA was subsequently purified using AMPure beads as described above. The DNA was subsequently tagged with ARP as described above.

### Library preparation and sequencing

Both the damage-enriched and input DNA were in vitro repaired using PreCR (NEB catalog number M0309L). The input DNA and supernatant of the pulldown were purified using AMPure beads. The purified pulldown was recombined with the beads, and library preparation was performed on the re-pooled sample containing the supernatant and the beads. A 125-bp paired-end ChIP-Seq library preparation kit (KAPA Biosystems catalog number KK8504) was used and sequencing performed using an Illumina HiSeq 2000 on first a rapid and then a high-output run (catalog number FC-401-4002). The resulting data were subsequently combined.

### Read processing library normalization and damage quantification

Unless stated, data processing was performed using R 3.4.0 and Bioconductor 3.5.

The quality of damage-enriched AP-seq samples (*n*=12) and corresponding input samples were checked using FastQC (https://www.bioinformatics.babraham.ac.uk/projects/fastqc/); the quality was sufficient that no further filtering was required before alignment. The reads were mapped to the reference human genome (version hg19) using the Bowtie2 algorithm (http://bowtie-bio.sourceforge.net/bowtie2/index.shtml) [84] with standard settings, allowing for two mismatches and random assignment of non-uniquely mapping reads. Mapping statistics are depicted in Fig. 3a. To confirm the robustness of key results, analyses were repeated excluding read duplicates and reads below mapping quality 10 (reads were filtered for mapping quality using SAMtools; http://www.htslib.org [85]). Data were visualized with the Integrative Genomics Viewer version 2.3.92 (http://software.broadinstitute.org/software/igv/) [86].

Paired reads were imported into R using the “GenomicAlignments” and “rtracklayer” [87] packages. Paired reads mapping more than 1-kb apart were discarded. The resulting median fragment length turned out to be < 250bp for AP-Seq (+input) and > 250bp for OGG1-AP-Seq (+input) samples (see Additional file 1: Figure S3B), despite the samples being processed together. Filters were applied to assess read duplication with Picard tools (https://broadinstitute.github.io/picard/), reads mapping to the Broad Institute blacklist regions (https://personal.broadinstitute.org/anshul/projects/encode/rawdata/blacklists/wgEncodeHg19ConsensusSignalArtifactRegions.bed.gz) [88], and whether reads overlap with repeats annotated in the UCSC RepeatMasker track from the UCSC Table Browser (rrmsk_hg19.bed). The main analysis was performed without applying these filters, but the robustness of key results was confirmed by repeating analyses with the filters.

Inter-library normalization was performed using only genomic areas of low damage. It was necessary to consider that increased exposure to DNA damage leads to increased library sizes. A global scaling factor was calculated as the mean read coverage in a low-damage subset (10%) of 100-kb bins, which were identified by their read coverage as the lowest decile of 100-kb bins over the mean of all samples.

Relative Enrichment of DNA damage was assessed through the normalised log2 fold change of the enriched sample over input (termed Relative Enrichment). This should account for biases derived from DNA amounts after genomic DNA extraction, as well as GC content biases from sequencing, which would affect the pull-down samples and inputs alike. Analyses were restricted to chromosomes 1 to 22 and X, except for the 100-kb damage distribution map which includes the Y chromosome (Fig. 1b).

All analyses were performed using the average Relative Enrichment in appropriate bin sizes tiled across the genome or covering genomic elements. For a large-scale overview, a bin size of 100-kb was chosen for comparability with related studies [28, 29]. Genome browser images were generated using absolute read counts pooled over replicates. Peak calling was generally not performed as it was deemed inappropriate for this type of data.

Each treatment condition was independently used for relative comparison within the samples. Lack of absolute quantification and subtle differences in fragment length suggest that instead of using primary AP-sites as input for OGG1-enriched AP-sites, it is more appropriate to show them side-by-side for comparison for those analyses that suggest subtle to no differences in distribution patterns. Sample-to-sample comparisons were limited to those analyses that show distinct differences in distribution patterns, such as G-quadruplexes, simple repeats, and telomeres.

Correlation of biological replicates was assessed using Pearson correlation in 100-kb resolution (Additional file 1: Figure S3).

### Analysis on local oxidative damage distribution

The karyogram map was compiled using the mean of the replicates at 100-kb resolution with “ggbio” [89] karyogram plot fixing the color scale to a Relative Enrichment of − 1 to 1. Enrichment over chromosomes was also depicted with 100-kb resolution for the mean of the replicates with shades depicting the standard error of the mean of triplicates. For illustration purposes, data were smoothed with a Gaussian smooth over 10 bins, using the smth.gaussian function of the “smoother” package. Correlations at 100-kb resolution were performed using Spearman correlation. Fine-resolution images were depicted using the IGV browser without any additional smoothing applied.

### Epigenome and feature analysis

Genome-wide feature sets were obtained from the UCSC Genome Browser. Chromatin features for HepG2 cells were retrieved from the data repository generated in the context of the ENCODE consortium and obtained through https://www.encodeproject.org/ [88]. Where applicable, datasets were pooled. Accession numbers are listed below.

Transcript density was calculated through the genome coverage with any one transcript as defined by UCSC. Distance to telomeres and centromeres was calculated as the absolute base pair distance to annotated telomeres and centromeres.

Genomic and chromatin features were calculated as mean values in 100-kb bins over the genome and clustered using hierarchical clustering of Spearman’s correlation coefficients. Features were then correlated (also Spearman) to the individual DNA damage levels. Data points represent the mean of the correlation coefficients with the standard error of the mean over replicates.

### GC content analysis

GC content analysis for quality control purposes was performed with the Deeptools suite [90] using default parameters. Visualization was performed in R using ggplot2. The range for GC content bins was limited to 20–70% GC content.

GC content preference of DNA damage distribution was assessed at 1-kb resolution. For each 1-kb bin in the genome, GC content was calculated and rounded to the closest percentage. Bins with more than 10% undefined sequence were censored. For all bins falling into a particular percentage range, mean Relative Enrichment was calculated with standard error of the mean for three biological replicates. Averaging over the bins in each category accounts for the lower numbers of bins with extreme GC content. For display purposes, a Gaussian smooth was applied reaching over 10% GC content range.

### DNA damage distribution over gene profile

Metaprofiles over coding genes were compiled using the UCSC transcript annotation. The mean was taken for different elements of genes, which are comprised of a total of 26,860 transcripts. Gene elements were either centered around an appropriate center point, in which case the mean Relative Enrichment was calculated for each base pair in the respective region. For gene elements of different sizes, the mean over the gene element was taken. Independent of their size, they were weighted as equal in subsequent analyses. The metaprofile was then compiled with the different gene elements in the following order: 48,838 promoters were centered around the transcriptional start site with 1-kb sequence in 5′ direction and 500 bp in 3′. 58,073 5′ UTRs, 214,919 exons, and 182,010 introns were addressed as a scaled mean. In addition, exons and introns were addressed through the exon-intron junction, both 5′ and in 3′ of the exon ± 250 bp. Given the small sizes of exons, 250 bp partially also contains following gene elements. The end of genes is represented through the means of 28,590 3′ UTRs and 43,736 transcription termination sites with 500 bp in 5′ direction and 1-kb in 3′. Twenty-two thousand four hundred eighty intergenic regions were addressed as the mean of each region. Shades represent the standard error of the mean over biological replicates.

Mean GC content distribution was determined using the same regions. Metaprofiles for GC content were smoothed using a Gaussian smooth over 100 bp.

### GC content- and transcription-dependent promoter, exon, and *Alu* analysis

Gene transcription was assessed using RNA-Seq data for HepG2 cells from the ENCODE consortium (Additional file 2: Table S2). Replicates were pooled, and RNA-Seq coverage was calculated for each unique UCSC-defined transcript (*n* = 57,564), normalized by the length of UTRs and exons. Promoters, i.e., the transcriptional start sites ± 1 kb for each transcript, were grouped into 11,058 silent promoters and the remaining 46,506 into deciles of increased transcriptional use. In parallel, the mean GC content for each promoter was calculated, which were then also grouped into deciles based on their GC content. Mean damage was assessed for each promoter in these groups.

Analysis of damage in exons was restricted to exons between 50 and 200 bp in size (*n* = 137,524) to avoid artifacts due to extreme sizes. RNA-Seq coverage and GC content were determined for each exon separately. Exons were then grouped into 48,706 silent exons and the remaining 88,818 grouped into deciles of increased exon expression. Equally, the exons were grouped into deciles.

*Alus* were also analyzed for GC content dependence, though not for expression, because instead of RNA-seq, a method of nascent transcription would be required for such an analysis. *Alus* were only considered when between 270 and 330 bp in size and intragenic to avoid artifacts through eu- and heterochromatic location. These 201,582 *Alus* were then grouped into GC content deciles and assessed for AP-site enrichment as described above.

Whole transcripts were also considered to be analyzed in a similar way. However, GC content of transcripts is highly dependent on transcript length, exon, and *Alu* density. Therefore, an analysis of the elements separately was deemed more appropriate.

### Retrotransposon analysis

Retrotransposon information was obtained from the UCSC repeat masker. For repetitive sequences, there is a risk of mapping issues and errors of annotation. Therefore, retrotransposon analysis was limited to families of these repeats, where location issues should not arise and misestimation of total repeat numbers should largely be balanced out through the pulldown vs. input comparison. Analyses for particular locations were restricted to the shorter *Alu* repeats, where mapping issues should be minimal, and the findings were confirmed by excluding ambiguous mapping.

*LINE* elements were defined as belonging to *LINE* element families of *L1PA7* or newer and only considered if between 5.9 and 6.1-kb (*n* = 2533) in size. *Alus* were considered when 270 to 330 bp in size (*n* = 848,350). Retrotransposons were anchored to their start sites and addressed with flanking regions from the start − 1 kb to + 7 kb for *LINE* elements and − 200 bp to + 500 bp for *Alu* elements. Metaprofiles were compiled as the mean Relative Enrichment over the respective region. GC content was assessed as the mean GC content at the particular site and smoothed using Gaussian smoothing in windows of 5% of feature length.

### Transcription factor binding sites, CpG islands, and G-quadruplex structure analysis

Transcription factor binding sites were obtained as the consensus set from ENCODE (Additional file 2: Table S2), which is cell line unspecific (*n* = 5,717,225). HepG2 cell-specific CTCF binding sites (*n* = 48,671) and DNase hypersensitivity sites (*n* = 192,735) were obtained through ENCODE and UCSC, respectively (Additional file 2: Table S2). G-quadruplex (G4) structures were obtained using the G4Hunter method [91], utilizing directly the reference file QP37_hg19_ref.RData provided with the associated R package (*n* = 359,446) with the exception of telomeric G4 structures with the center less than 500 bp away from the chromosome end (*n* = 3). CpG islands were defined through UCSC (*n* = 27,443). Features were considered to be in a promoter, if they overlap with the region of a transcriptional start site ± 1 kb. They were considered to overlap with DNase hypersensitivity only when the feature itself overlaps with a DNase hypersensitivity site. Transcription factor binding sites were excluded, if located within 500 bp of the center of a CTCF binding site. For metaprofiles, the centers of the features were considered and mean Relative Enrichment of damage levels assessed relative to the center point. For quantification of mean damage at a given feature site, only the feature itself was addressed and quantified as the mean Relative Enrichment over the region. GC content over transcription factor binding sites was however calculated as the mean over the region around the transcription factor binding site (± 500 bp). Groups of features were summarized using the median.

### Telomere analysis

Due to expected mapping artifacts at telomeric repeats, telomeres were addressed separately not using the aligned sequence. Instead, TelomereHunter version 1.0.4. (https://www.dkfz.de/en/applied-bioinformatics/telomerehunter/telomerehunter.html) [92] was used to filter out reads that map to telomeric repeats. These were reassigned to intratelomeric and subtelomeric regions or other locations. Of these, only the intratelomeric repeats were considered. Normalization between libraries was performed not within the TelomereHunter package but separately with the global scaling factor as described above using only genomic areas of low damage accumulation. The global scaling factor was calculated as the mean read coverage in a low-damage subset (10%) of 100-kb bins, which were identified by their read coverage as the lowest decile of 100-kb bins over the mean of all samples. Mean Relative Enrichment between biological replicates was calculated with the standard error of the mean.

### Microsatellite analysis

Microsatellites were defined through the UCSC repeat masker as the “Simple_repeat” class. For quantification purposes, reverse complement repeat classes were combined. Only microsatellite sequences that are represented > 1000 times in the genome were considered. This leaves 39 repeat types, which represented by a total of 388,350 repeats. Since the damage assessment does not allow strand specificity, repeats were pooled with their reverse complement assigning both orientations to the alphabetically first repeat. Median Relative Enrichment of damage was quantified over each microsatellite type.

### Chromatin loop definition

Chromatin loop anchor definition was inspired by Canela et al. [93] using the overlap of CTCF binding sites (*n* = 48,671) with RAD21 binding sites (*n* = 64,528). In addition, we included SMC3 binding sites (*n* = 30,782). Each binding site was defined by ChIP-seq in HepG2 cells obtained from ENCODE (Additional file 2: Table S2). CTCF sites are only considered, if they overlap with a canonical CTCF motif (*n* = 33,692) as defined by the package “motifmatchr.” Loop anchors were centered and oriented at the center of the motif but merged and recentered, if closer than 500 bp apart. The resulting chromatin loops (*n* = 18,242) were then oriented by the direction of the CTCF motif. Coverage with the original ChiP-seq signal of the three components was assessed through mean coverage profiles of the original coverage tracks from ENCODE (Additional file 2: Table S2). These data were not normalized or corrected, as it was not deemed necessary for the assessment of relative coverage. Damage distribution analysis around loop anchors was performed as described in 8.12 using loop anchors ± 500 bp.

### Chromatin loop insulation classification

Chromatin loops were assessed for their insulation properties regarding changes between open and closed chromatin inside and outside of the loop. As markers of active and inactive chromatin, the log2 ratios of H3K36me3 and H3K27me3 read coverage 10-kb outside and inside the loop was determined. Their ratio was used to assess the insulation properties. Based on the distribution of this ratio, an otherwise arbitrary cut-off of 1.2 was used to separate out those loops that display clear changes from H3K36me3 to H3K27me3, i.e., “swap OFF” (*n* = 1767), and those that change from H3K27me3 to H3K36 me3, i.e., “swap ON” (*n* = 2021). The remaining loop anchors were then differentiated dependent on whether H3K27me3 or HeK36me3 is the dominant histone mark, determined on whether log2(H3K27me3/H3K36me3)<2, defining loops in chromatin stated as “ON” (*n* = 3975) and loops > 2 stated as “OFF” (*n* = 10,479). Chromatin loops defined as “OFF” can therefore also be located in heterochromatin. Chromatin changes were confirmed by determining the mean coverage distribution of the raw read coverage over the defined groups.

### Chromatin architecture-dependent oxidative damage assessment

AP-seq enrichment was determined as described in 8.12. in the region ± 10 kb from the loop anchor. For differential changes in damage levels, AP-seq Relative Enrichment was determined and the differential of the 10-kb inside and outside the loop. Statistical testing to determine the differential damage enrichment between the “swap ON” and “swap OFF” group was performed with the Wilcoxon ranked sum test.

### Patient selection for mutation analysis

Data for mutations in cancer were obtained from the Pan-cancer Analysis of Whole Genomes consortium [70]. Contributions of mutational signatures were provided by PCAWG working group 7 [74].

The dataset is comprised of 2702 tumor-normal pairs for 39 cancer types. From this dataset, we obtained all data on mutation rates and mutation signature contributions, as well as clinical metadata. The analysis was restricted to chromosomes 1 to 22 and X. It was focused on C-to-A and T-to-G mutations as these are the major mutation types derived from oxidative damage—C-to-A from oxidative damage in the genome and T-to-G from incorporation of oxidized nucleosides during replication. In addition, we investigated the mutation patterns for C-to-A mutations under conditions of POLE proofreading-deficient tumors. These mutations are suspected to arise in a yet unknown proportion from mismatches with oxidatively damaged DNA [71]. For control purposes on POLE proofreading-deficient tumors, C-to-T mutations were included. Equally prominent as C-to-A mutations, their underlying biology is largely unclear but suspected to arise from bypass of uracil and direct mispairing of C-A pairs [77, 78]. The involvement of base excision repair in removal of uracils therefore suggests partially overlapping biological mechanisms. These mutation types include the respective reverse complements G-to-T, A-to-C, and G-to-A, as the analysis is not performed strand specifically. Effects from selection processes were not taken into consideration, because the consequences from the average 2.9 driver SNVs per tumor [94] on the mutation patterns should be negligible.

Patients with oxidative damage-induced mutations were separated based on the proportion contribution of Signature 18 to C-to-A mutations and by the contribution of Signature 17 to T-to-G mutations. Patients were censored that have a hypermutator phenotype (C-to-A > 100,000; *n* = 9) or coding mutations in 8-oxoG or AP-site processing, i.e., whether mutations fall into the coding sequence of OGG1 (*n* = 7), APEX1 (*n* = 3), or FEN1 (*n* = 3). Mutations were considered, if their effect determined by the ensembl VEP tool (http://www.ensembl.org/Multi/Tools/VEP) [95] identified them as missense variants, stop codon gained, frameshift variants, or splice donor variant. Copy number alterations were not considered. For information of individual patients, see Additional file 2: Table S2. In addition, patients were also censored based on documented smoking history or previous exposure to chemotherapy/radiotherapy. A total of 2401 samples were used for analysis. They were grouped into Signature 18-based groups of < 10% (*n* = 1398), 10 to 40% (*n* = 322), 40 to 60% (*n* = 540), and > 60% (*n* = 141). Based in Signature 17, they were grouped into < 10% (*n* = 2255), 10 to 25% (*n* = 78), 25 to 50% (*n* = 59), and > 50% (*n* = 9).

Patient samples with a polymerase epsilon proofreading defect (*n* = 8) were determined through a hypermutator phenotype (C-to-A > 100,000) with prominence of Signature 10 confirmed as being linked to coding mutations in Pol E. In total, these samples contain 3,436,531 C-to-A mutations. For information of individual patients, see Additional file 2: Table S3.

### GC content preferences of mutation rates

For each 1-kb bin in the genome, GC content was calculated and rounded to the closest percentage. Bins with more than 10% undefined sequence were censored. Mutations falling into bins of 50% GC content or higher were calculated as proportion of the total C-to-A and T-to-G mutation counts (drawing the cut-off at 60% GC content gives equivalent results). Assuming equal distribution dependent exclusively on base content, a total of 15% of C-to-A and 8% of T-to-G mutations would be expected to fall into such high GC content areas of the genome. However, in the case of C-to-A mutations, even in the control tumor samples with Signature 18 proportion < 0.1, only a median of 9% C-to-A mutations fall into high GC content. The cut-off was determined based on the observed AP-site distribution patterns. Statistic testing was performed relative to the control groups of < 0.1 signature contribution using the Wilcoxon ranked sum test.

High GC content DNA is associated with replication timing. Therefore, the genome was separated into early, intermediate, and late replicating DNA, based on Repli-Seq data on HepG2 cells (see Additional file 2: Table S2). One-kilobyte bins were separated into tertiles of replication timing and mutation rates in high GC content DNA assessed separately within these groups. High GC content DNA is overrepresented in early replicating DNA (807 Mb) vs. intermediate (78 Mb) and late replicating 1-kb bins (24 Mb). To account for this bias, GC content-dependent mutation rates were assessed separately in all three groups for the mutation-rich POLE proofreading-deficient tumor samples.

### Genomic features analysis

Metaprofiles over genomic features were calculated for the features with the same selection strategy as described above. For this, mutations of each mutation type were pooled for each patient group. Mean relative mutation rates over features were calculated as relative C-to-A or C-to-T mutation density normalized to 1,000,000 C-to-A or C-to-T mutations per patient group. The mean over the features was normalized for sequence content of the particular location by dividing with a factor of the local GC content divided by the average of 41%. For display purposes, data were smoothed using a Gaussian smooth spreading over 100 bp for the gene body profile, *Alus*, protein-binding sites, CpG islands, and G4 structures. *LINE* elements were smoothed using Gaussian smoothing over 200 bp to account for the increased noise originating from the lower frequency of this particular feature.

### Reanalysis of the study by Ding et al.

#### Data processing

Raw data were obtained from NCBI BioProject accession number PRJNA359996 and quality controlled using FastQC (https://www.bioinformatics.babraham.ac.uk/projects/fastqc/). Reads were aligned to the mouse genome (mm10) using Bowtie2 with default parameters. Because differences in library size can affect the downstream analyses, libraries were also subsampled with samtools [85] to 42,908,708 read pairs, the library size of mapped reads of the wildtype (WT) sample.

For easier reproducibility, analyses on GC bias, gene metaprofiles, and the generation of genome browser tracks was performed with the Deeptools suite [90].

### GC sequencing bias

GC content was assessed with Deeptools on the size-corrected libraries using default parameters. Visualization was performed in R using ggplot2. The range for GC content bins was limited to 20–70% GC content.

### Gene profile and genome browser tracks

Gene profiles were produced with the Deeptools suite and default normalization settings on the size-corrected libraries. Gene bodies were scaled to 5-kb and supplemented with 3-kb from the transcript start and end. Bigwig files were produced with 100-bp binning and visualized with the Integrative Genomics Viewer version 2.3.92 (http://software.broadinstitute.org/software/igv/) [86]. The repeat masker and CpG island tracks were obtained from the UCSC genome browser.

### Peak calling and processing

Peak calling was performed using MACS2 (https://github.com/taoliu/MACS/) with default settings, both on the original library size and subsampled to 42,908,708 read pairs. Peaks were filtered for > 3-fold, > 4-fold, and > 5-fold enrichment, for which the numbers were comparable with Ding et al. both for the approaches on the full library sizes and with subsampled libraries. Peaks were further processed using the “GenomicRanges” package in R. Genomic annotation of peaks was determined using the “ChIPSeeker” package [96] with the UCSC mm10 transcript library. Repeat annotation was obtained from the UCSC repeat masker. Simple repeats were combined with their reverse complement and assigned alphabetically.

### Trinucleotide distributions over the genome

Trinucleotide frequencies were assessed through assignment of each 1-kb bin in the hg19 genome to a GC content category. Reverse complement trinucleotides were combined, and the proportion of each of the 32 sequences was calculated separately for each GC content category. In addition, GC contents above 50% were combined. To account for total frequencies of trinucleotides throughout the genome, it was assessed which proportion of the total trinucleotide counts falls into GC content of > 50%, which accounts for 10.9% of the genome.

Trinucleotides that underlie the mutations associated with mutational signatures for a specific mutation type were proportionally added to account for the fingerprint of the signature. It was calculated which proportion of these mutations fall into particular GC content category. This calculation is based on sequence content alone and does not account for epigenetic confounders, e.g., high GC content DNA is enriched in euchromatic domains and early replicating DNA, which generally show lower mutation rates than heterochromatin. We used these data as controls to highlight possible biases, such as the probability to call Signatures dependent on where the mutations locate. These data were not used to correct mutation data, since the sequence content is expected to impact the underlying biology, such as damage impact and repair efficiency. A trinucleotide-based sequence content correction was therefore considered to compromise the comparability between the data on mutations and DNA damage distribution. Consequently, trinucleotide-based data correction was not applied in the context of this paper.

## Additional file

Additional file 1: **Figure S1.** Schematic diagram of the chemical enrichment process of AP-sites using an aldehyde reactive probe. **Figure S2.** Quality control measures for successful treatment and pulldown specificity. **Figure S3.** Sequencing statistics for AP-seq. **Figure S4.** Additional data for damage distribution including all treatment conditions. **Figure S5.** Additional data on GC content, transcription, or accessibility dependence. **Figure S6.** Reanalysis of the analysis by Ding et al. **Figure S7.** SNVs from POLE proofreading-deficient cancers follow distinct patterns. **Figure S8.** SNVs from POLE proofreading-deficient cancers spare out high GC content DNA, irrespective of replication timing. **Figure S9.** Trinucleotide distribution in high GC content DNA. (PDF 9209 kb)

Additional file 2: **Table S1.** Oligonucleotides and primers used for in vitro pulldown experiments. **Table S2.** Selected tumor samples with coding mutations in OGG1, APEX1, of FEN1. **Table S3.** Selected tumor samples with polymerase epsilon proofreading defect. (DOCX 17 kb)
