# Supplementary material for: Genomic landscape of oxidative DNA damage and repair reveals regioselective protection from mutagenesis
Source: Genome Biol. 2018 Dec 7;19:215. doi: 10.1186/s13059-018-1582-2 (PMC6284305; doi:10.1186/s13059-018-1582-2)
Supplement: Supplementary file 1 — Figure S1. Schematic diagram of the chemical enrichment process of AP-sites using an aldehyde reactive probe. Figure S2. Quality control measures for successful treatment and pulldown specificity. Figure S3. Sequencing statistics for AP-seq. Figure S4. Additional data for damage distribution including all treatment conditions. Figure S5. Additional data on GC content, transcription, or accessibility dependence. Figure S6. Reanalysis of the analysis by Ding et al. Figure S7. SNVs from POLE proofreading-deficient cancers follow distinct patterns. Figure S8. SNVs from POLE proofreading-deficient cancers spare out high GC content DNA, irrespective of replication timing. Figure S9. Trinucleotide distribution in high GC content DNA. (PDF 9209 kb) [file 13059_2018_1582_MOESM1_ESM.pdf]

Supplemental Material for:

Genomic landscape of oxidative DNA damage and repair reveals regioselective protection from mutagenesis

Anna R Poetsch[1,2,3,*], Simon J Boulton[1,†], Nicholas M Luscombe[1,2,3,†]

[1] The Francis Crick Institute, 1 Midland Road, London, NW1 1AT, UK
[2] Okinawa Institute of Science & Technology Graduate University, Okinawa 904-0495, Japan
[3] UCL Genetics Institute, University College London, Gower Street, London WC1E 6BT, UK

*Corresponding author: arpoetsch@gmail.com

† These authors contributed equally

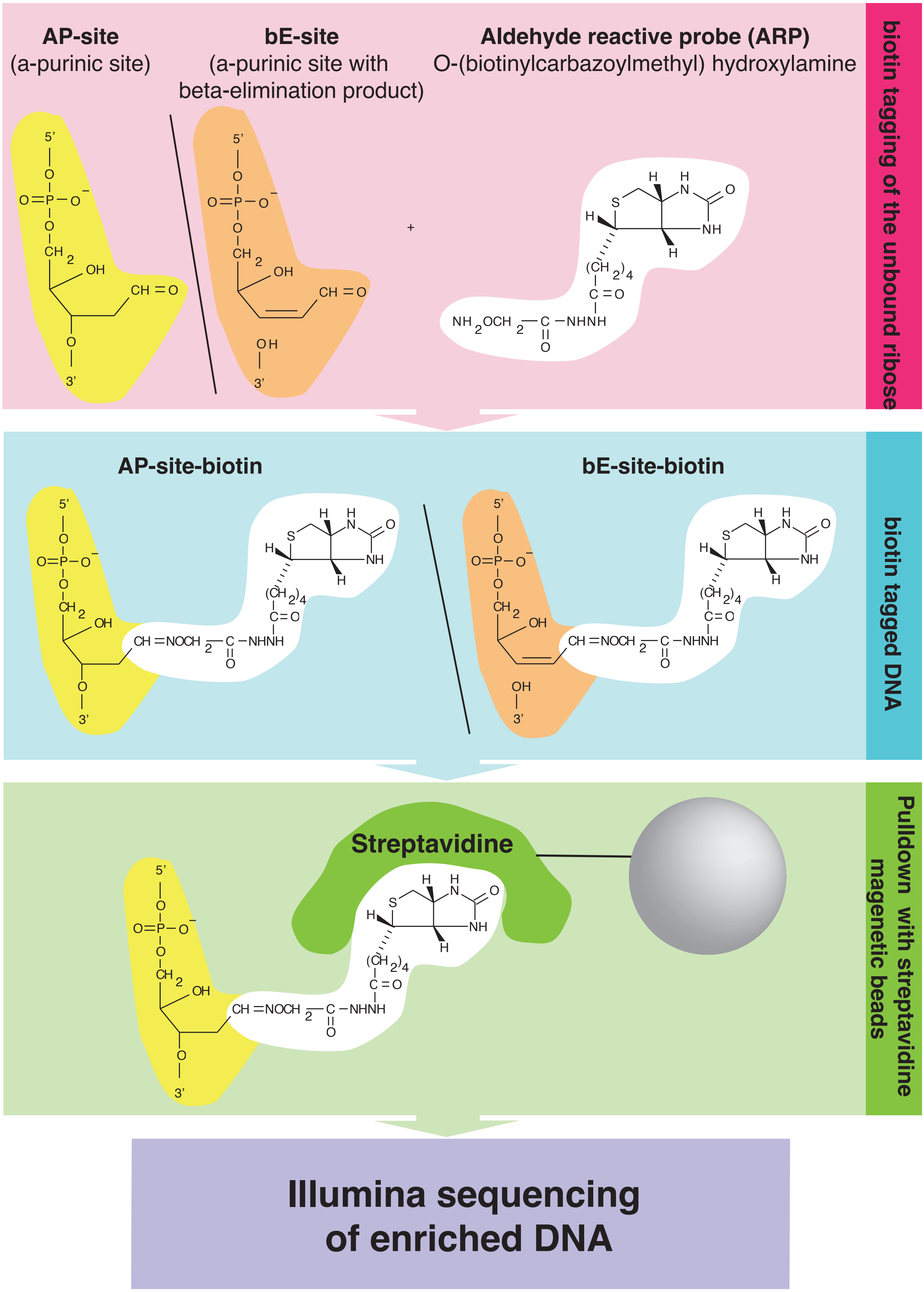

Fig. S1. Schematic diagram of the chemical enrichment process of AP-sites using an aldehyde reactive probe. AP-sites and the beta-elimination intermediates of AP-sites are biotin-tagged using an aldehyde reactive probe (ARP) on the damaged strand. Subsequently they are fragmented, and the double stranded DNA is pulled-down with streptavidin. The enriched DNA is processed for sequencing and mapped to the reference genome.

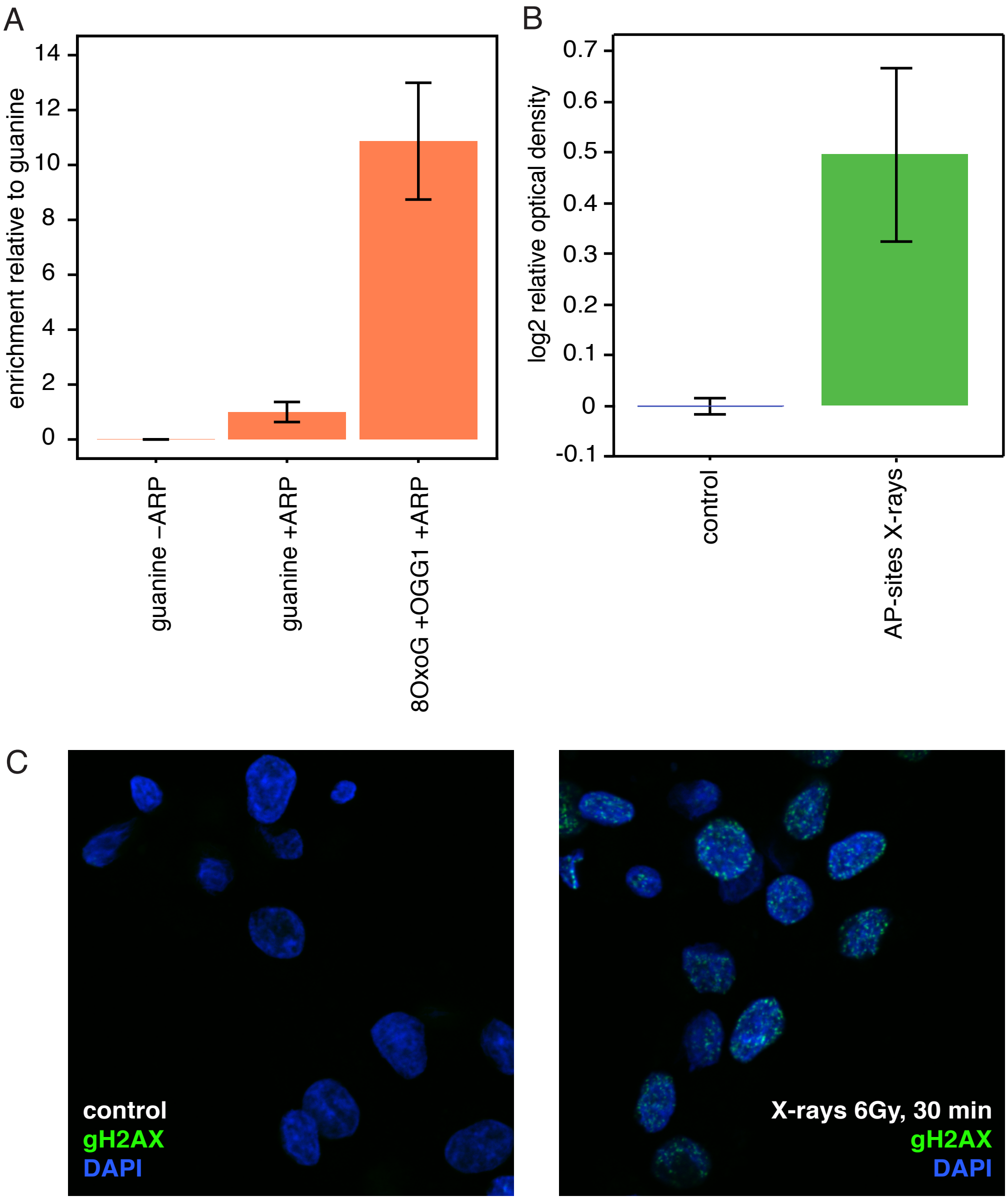

Fig. S2. Quality control measures for successful treatment and pulldown specificity. **(A)** *In vitro* pulldown with the Aldehyde Reactive Probe (ARP) of standardized oligonucleotides. Oligonucleotides containing 8-oxoG converted into an AP-site were pulled down *in vitro* using guanine as control. The DNA was treated in triplicates with OGG1+ARP, ARP alone or was not biotin-tagged. The efficiency of the pulldown was determined as recovery of input and normalised to the guanine control that represents the background levels arising from spontaneous AP-site formation and unspecific probe reaction. Pulldown of untagged DNA is negligible. Pulldown of AP-sites that are created with OGG1 digest of 8-oxoG are recovered ~10-fold over undamaged oligonucleotides, which is significant ($p<0.05$, student's t-test). Depicted is the mean and standard error of the mean. **(B)** Colorimetric measurement of AP-sites after X-ray treatment (30 min, 6Gy) using the Aldehyde Reactive Probe. Data are quantified in triplicates as the log2-fold change of normalized optical density relative to the untreated control ($p<0.05$, student's t-test). Depicted is the mean with standard error of the mean. **(C)** Immunofluorescence staining of γH2AX (green) as a measure of radiation induced DNA damage and nuclear staining as a reference (blue) under untreated conditions (left) and after treatment with 6Gy X-rays and 30min incubation (right).

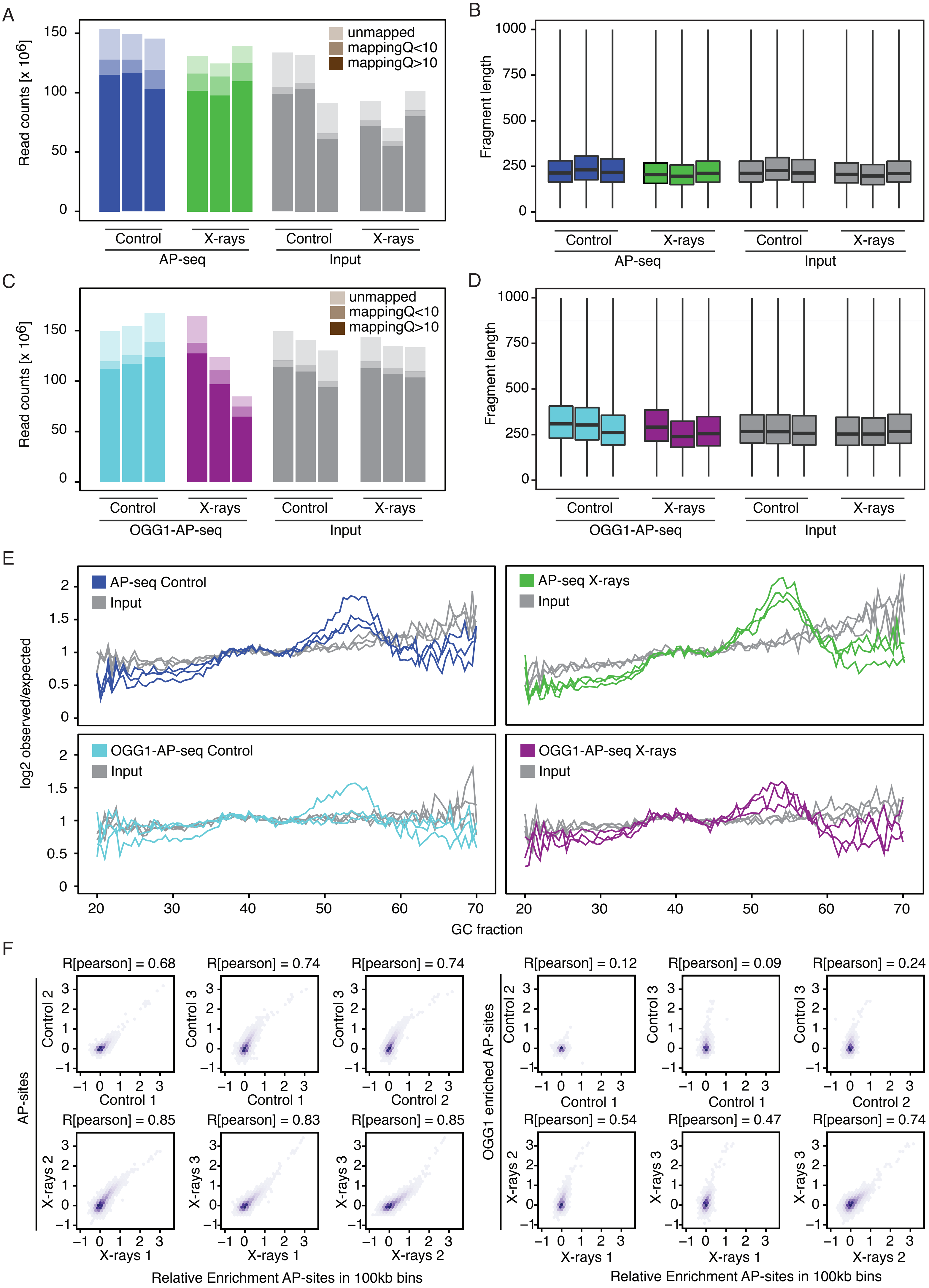

A
Read counts [x 10^6]
unmapped
mappingQ<10
mappingQ>10
Control
X-rays
AP-seq
Input
B
Fragment length
C
OGG1-AP-seq
D
E
AP-seq Control
AP-seq X-rays
OGG1-AP-seq Control
OGG1-AP-seq X-rays
log2 observed/expected
GC fraction
F
R[pearson] = 0.68
R[pearson] = 0.74
R[pearson] = 0.74
R[pearson] = 0.12
R[pearson] = 0.09
R[pearson] = 0.24
R[pearson] = 0.85
R[pearson] = 0.83
R[pearson] = 0.85
R[pearson] = 0.54
R[pearson] = 0.47
R[pearson] = 0.74
AP-sites
OGG1 enriched AP-sites
Control 1
Control 2
Control 3
X-rays 1
X-rays 2
X-rays 3
Relative Enrichment AP-sites in 100kb bins

Fig. S3. Sequencing statistics for AP-seq. **(A,B)** Read counts for each replicate differentiating reads that did not map to the human genome (hg19; light colour), reads that map with a mapping quality <10 (medium light colour), and reads that map with mapping quality >10 (dark colour). Mapping quality is a measure for non-unique sequences, so it can be observed that in the AP-Seq samples more reads are mapping ambiguously than in the input samples. **(C,D)** Read length of mapped reads for each replicate of AP-Seq (C)**,** OGG1-AP-Seq (D), and corresponding input samples depicted as box-plots. Throughout treatment conditions, read length ranges continuously around 250bp. Although treated in one experiment, median read length of OGG1-AP-Seq samples and their input is ~50bp longer than AP-Seq without modification. Therefore, direct comparison of samples was carefully controlled and only applied, if effects of resolution and normalisation could be excluded. **(E)** GC sequencing bias assessment of Input, and AP-Seq. Depicted is the log2 observed/expected ratio of read counts in bins from 20% to 70% GC content. In the input samples, read counts increase consistently with increased GC content. The AP-Seq samples display a more complex pattern of GC content differences, which reflects the underlying biology of this measurement. **(F)** Correlation of treatment replicates at 100kb resolution. Relative Enrichment was correlated for each replicate in 100kb resolution using Spearman correlation. Depicted is the relative density of the pairwise correlation for the relative enrichment in all four conditions. Correlation is dependent on non-random distinct distribution patterns. Therefore, the conditions differ in their correlation coefficients dependent on the distinctness of enrichment patterns, with AP-sites and X-ray treatment representing the highest correlation coefficients.

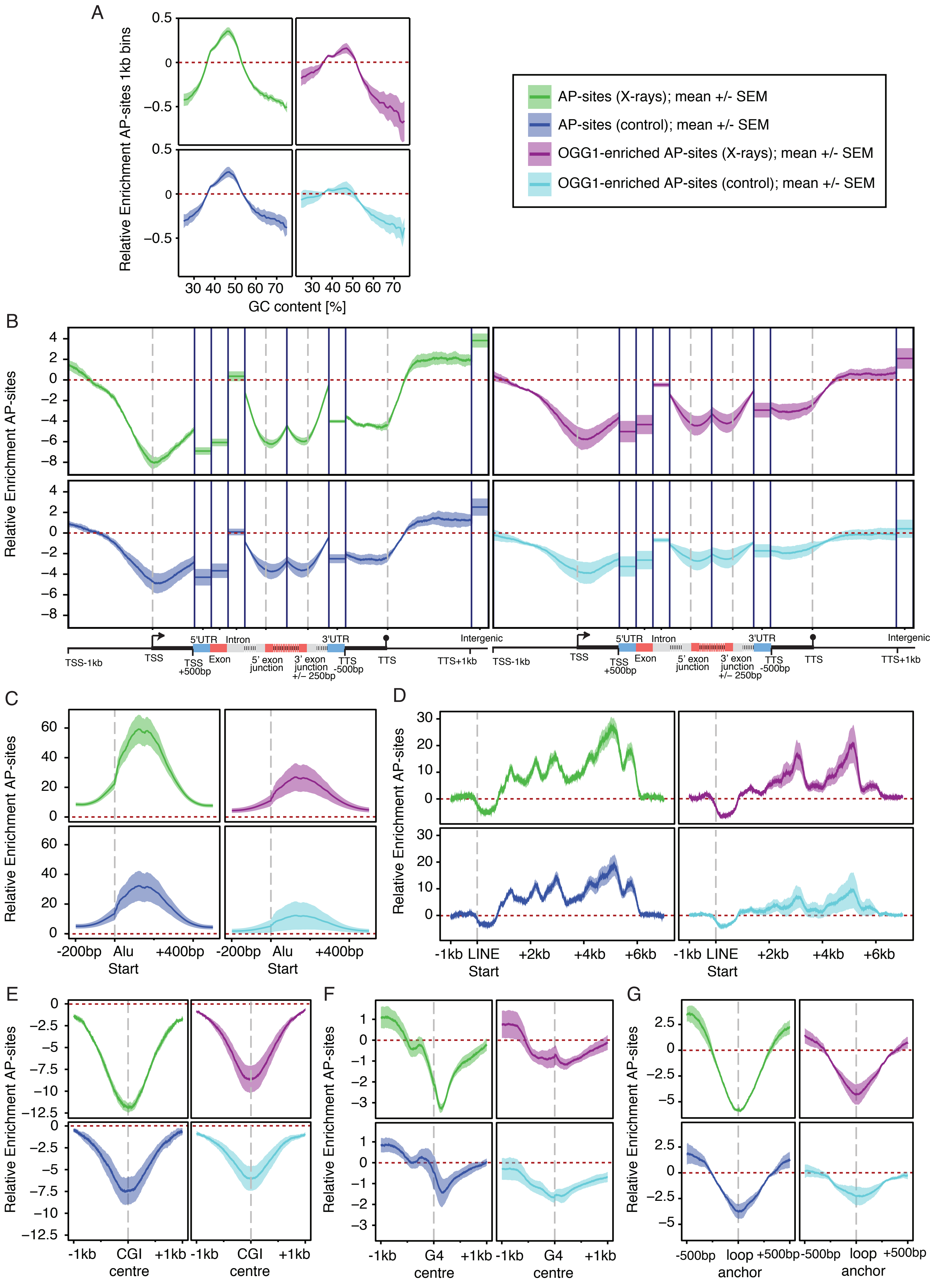
A
B
C
D
E
F
G
Relative Enrichment AP-sites 1kb bins
GC content [%]
AP-sites (X-rays); mean +/- SEM
AP-sites (control); mean +/- SEM
OGG1-enriched AP-sites (X-rays); mean +/- SEM
OGG1-enriched AP-sites (control); mean +/- SEM
Relative Enrichment AP-sites
TSS-1kb
TSS
TSS +500bp
5'UTR
Exon
Intron
5' exon junction
3' exon junction +/− 250bp
3'UTR
TTS -500bp
TTS
TTS+1kb
Intergenic
-200bp
Alu Start
+400bp
-1kb
LINE Start
+2kb
+4kb
+6kb
CGI centre
+1kb
G4 centre
-500bp
loop anchor
+500bp

Fig. S4. Additional data for damage distribution including all treatment conditions. Subtle differences in distribution patterns between treatment conditions suggest mostly non-locus-specific rapid processing of 8-oxoG into AP-sites, except for G-quadruplexes and the body of LINE-elements. **(A)** The plots show dependence between Relative Enrichment of damage and genomic GC content at 1kb resolution. For all treatment conditions, damage levels increase with GC content and then surprisingly fall in high GC areas. AP-site enrichment after X-ray treatment shows the most pronounced signal. **(B)** Metaprofile of Relative Enrichment over ~23,000 protein-coding genes (n_{genes}=23,056, $n_{promoters}$=48,838, n_{5UTRs}=58,073, n_{exons}=214,919, $n_{introns}$=182,010, n_{3UTRs}=28,590, $n_{termination}$=43,736, $n_{intergenic}$=22,480). Damage levels for UTRs, exons, introns, and intergenic regions are averaged across each feature due to their variable sizes. In all treatment conditions, coding and regulatory regions are depleted for damage despite their increased GC content, whereas introns have near intergenic damage levels. **(C)** Metaprofiles of Relative Enrichments across 848,350 *Alu* elements. There is a very large accumulation of damage inside these features, in particular for AP-site enrichment for X-ray treated samples. **(D)** Metaprofiles of Relative Enrichments across 2,533 *LINE* elements. For all treatment conditions, damage levels are strongly reduced in the *LINE* promoter region, but highly accumulated in the body of the line element. For *LINE* elements, AP-sites and OGG1-enriched AP-sites show similar quantitative range after treatment, the pattern themselves are however different, which may be prompted by the G-quadruplex forming ability of *LINE* elements. **(E)** Metaprofiles centred on CpG islands (n= 28,595). Damage levels are reduced in all treatment conditions. **(F)** Metaprofiles centred on predicted G-quadruplexes (n=359,449). There are asymmetrically reduced damage levels for AP-sites, but not for OGG1-enriched AP-sites, increased by treatment. **(G)** Metaprofiles centered about chromatin loop anchors (n=18,242). All treatment conditions show reduction of damage levels on loop anchors with a slower increase of damage towards the inside of the loop versus the outside of the loop. For all panels, the mean across three biological replicates is depicted and, as shaded borders, the standard error of mean.

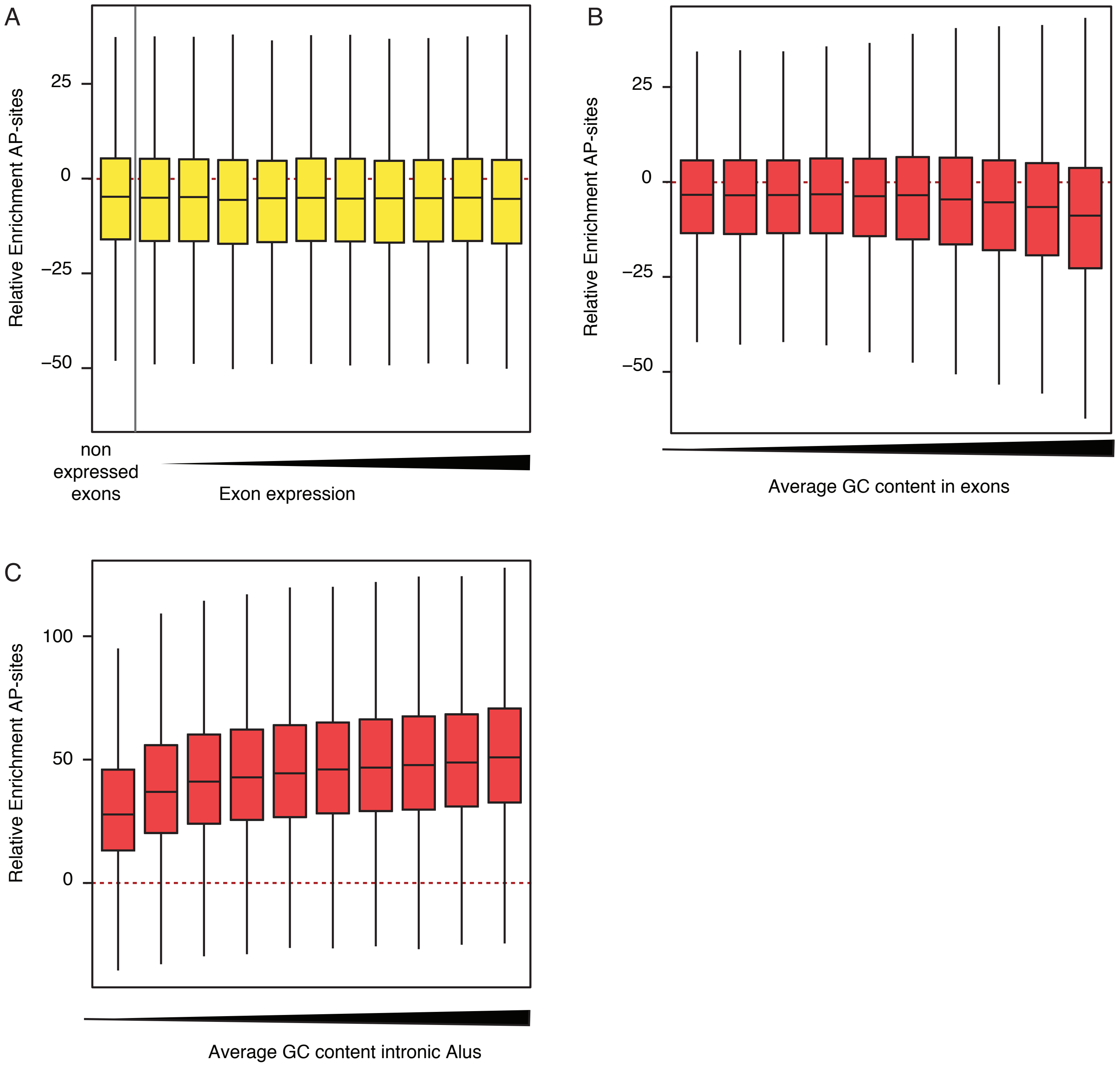

Fig. S5. Additional data on GC content, transcription, or accessibility dependence. **(A/B)** Boxplots depict damage levels at 214,919 exons binned into unexpressed and expression deciles (A), and average GC content deciles (B). Like in promoters, damage is not transcription-dependent, but reduces with increasing promoter GC content. **(C)** Boxplots depict damage levels dependent on GC content in 201,582 intronic *Alus* between 270 and 330bp in size. Damage enrichment increases with increased GC content. A correction for GC content was not applied.

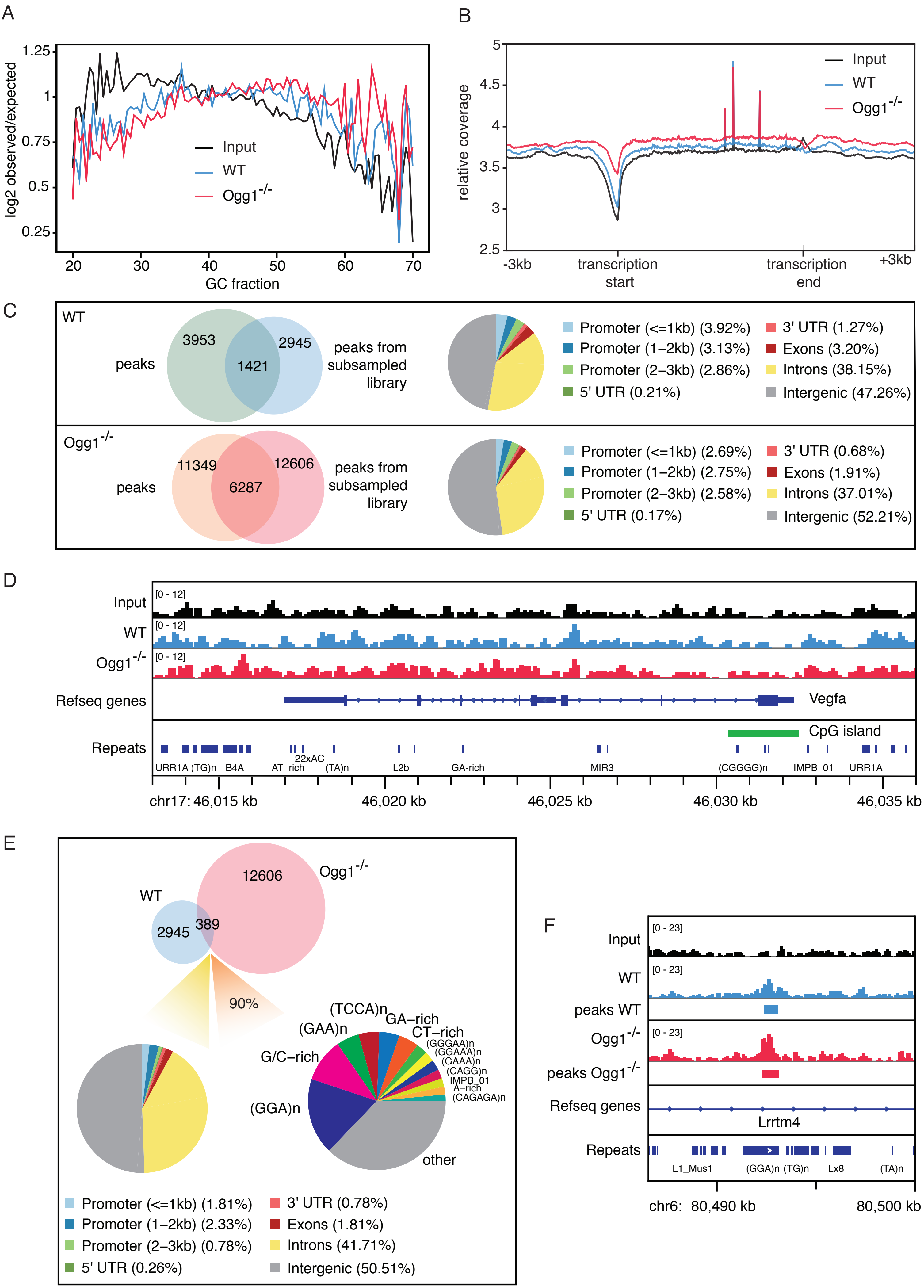

A
log2 observed/expected
GC fraction
Input
WT
Ogg1-/-
B
relative coverage
-3kb
transcription start
transcription end
+3kb
Input
WT
Ogg1-/-
C
WT
peaks
3953
1421
2945
peaks from subsampled library
Promoter (<=1kb) (3.92%)
Promoter (1–2kb) (3.13%)
Promoter (2–3kb) (2.86%)
5' UTR (0.21%)
3' UTR (1.27%)
Exons (3.20%)
Introns (38.15%)
Intergenic (47.26%)
Ogg1-/-
peaks
11349
6287
12606
peaks from subsampled library
Promoter (<=1kb) (2.69%)
Promoter (1–2kb) (2.75%)
Promoter (2–3kb) (2.58%)
5' UTR (0.17%)
3' UTR (0.68%)
Exons (1.91%)
Introns (37.01%)
Intergenic (52.21%)
D
Input
WT
Ogg1-/-
[0 - 12]
Refseq genes
Vegfa
CpG island
Repeats
URR1A (TG)n B4A AT_rich 22xAC (TA)n L2b GA-rich MIR3 (CGGGG)n IMPB_01 URR1A
chr17: 46,015 kb
46,020 kb
46,025 kb
46,030 kb
46,035 kb
E
WT
Ogg1-/-
2945
389
12606
90%
(TCCA)n
GA–rich
CT–rich
(GAA)n
G/C–rich
(GGGAA)n
(GGAAA)n
(GAAA)n
(CAGG)n
IMPB_01
A–rich
(CAGAGA)n
(GGA)n
other
Promoter (<=1kb) (1.81%)
Promoter (1–2kb) (2.33%)
Promoter (2–3kb) (0.78%)
5' UTR (0.26%)
3' UTR (0.78%)
Exons (1.81%)
Introns (41.71%)
Intergenic (50.51%)
F
Input
WT
peaks WT
Ogg1-/-
peaks Ogg1-/-
[0 - 23]
Refseq genes
Lrrtm4
Repeats
L1_Mus1 (GGA)n (TG)n Lx8 (TA)n
chr6: 80,490 kb
80,500 kb

Fig. S6: Reanalysis Ding et al. [36] The study by Ding *et al*. concludes that "Gene promoters and UTRs were found to harbor more OG-enriched sites than expected if the sites were randomly distributed throughout the genome". While we address AP-sites and AP-sites in combination with 8-oxoG, we find the opposite in our data, a protection of promoters and UTRs from oxidative damage, as do Wu *et al* [37] for measuring 8-oxoG directly in yeast. Therefore, we found it necessary to reassess the data analysis by Ding *et al* to clarify the status of oxidative damage in these functionally important genomic regions. This reassessment does not support the conclusions of increased oxidative DNA damage in promoters and 5' UTRs. We could however confirm the findings of 8-oxoG peaks in DNA with simple repeats. **(A)** GC sequencing bias assessment of Input, wildtype (WT), and Ogg$^{-/-}$. Depicted is the log2 observed/expected ratio of read counts in bins from 20% to 70% GC content. Reduced read counts of high GC content is strongest in the input sample. As this is the only sample used as control in peak calling, this difference in GC bias with reduced read counts in GC rich DNA leads to a bias in peak calling towards GC rich DNA. **(B)** Scaled metaprofile depicting the mean relative coverage of OG-seq reads over 63,759 mouse transcripts. Genes are scaled to length with 3kb added to the transcription start and end. Promoter regions are highly reduced in signal in all three samples, strongest in the Input, which reflects the GC sequencing bias. From this metaprofile it is not possible to conclude differential distribution of 8-oxoG over genes. **(C)** Peak calling has been performed using MACS2 with default settings on the original libraries, and after adjustment to equal library sizes to avoid library size biases during peak calling. Peak counts are depicted for peaks with at least 3-fold enrichment. Peak numbers are comparable with the original analysis by Ding *et al*. However, only about half of the peaks are reproducible for both approaches equally for WT and Ogg1$^{-/-}$, which suggests that these data are not suitable for a robust analysis using this type of peak calling. Annotation to genomic features of the peak group with adjusted library sizes shows that most peaks are located in intergenic and intronic DNA. 0.21% and 0.17% of peaks annotate to 5' UTRs, the published 2.4% and 1.6% cannot be reproduced. **(D)** Browser profile of the Vegfa gene to illustrate a typical enrichment profile on a biologically relevant example, as Vegfa is described to be regulated through 8-oxoG accumulation in the G4 structure that can form in the CpG island of its promoter. There is no indication of OG-seq enrichment in this area. Instead, all samples, including the input, present with reduced read counts in the GC rich promoter of Vegfa. **(E)** Of the peaks derived from subsampled libraries, the overlap between WT and Ogg1$^{-/-}$ is depicted. Annotation analysis of this peak group does also not suggest specific enrichments dependent on gene elements. However, 90% of the peaks overlap with repetitive DNA. Differentiation of these repeats reveals a dominance of (GGA)n, G/C-rich repeats, and (GAA)n. **(F)** Example locus of peaks within repetitive DNA showing signals in both the WT and Ogg1-/- samples, with a stronger signal in the latter, which was confirmed for several loci. Also Ding *et al*. Figure 2 represents such a locus. Therefore, the finding of increased 8-oxoG as measured with OG-Seq in repetitive sequences could be confirmed, with (GGA)n representing the largest group.

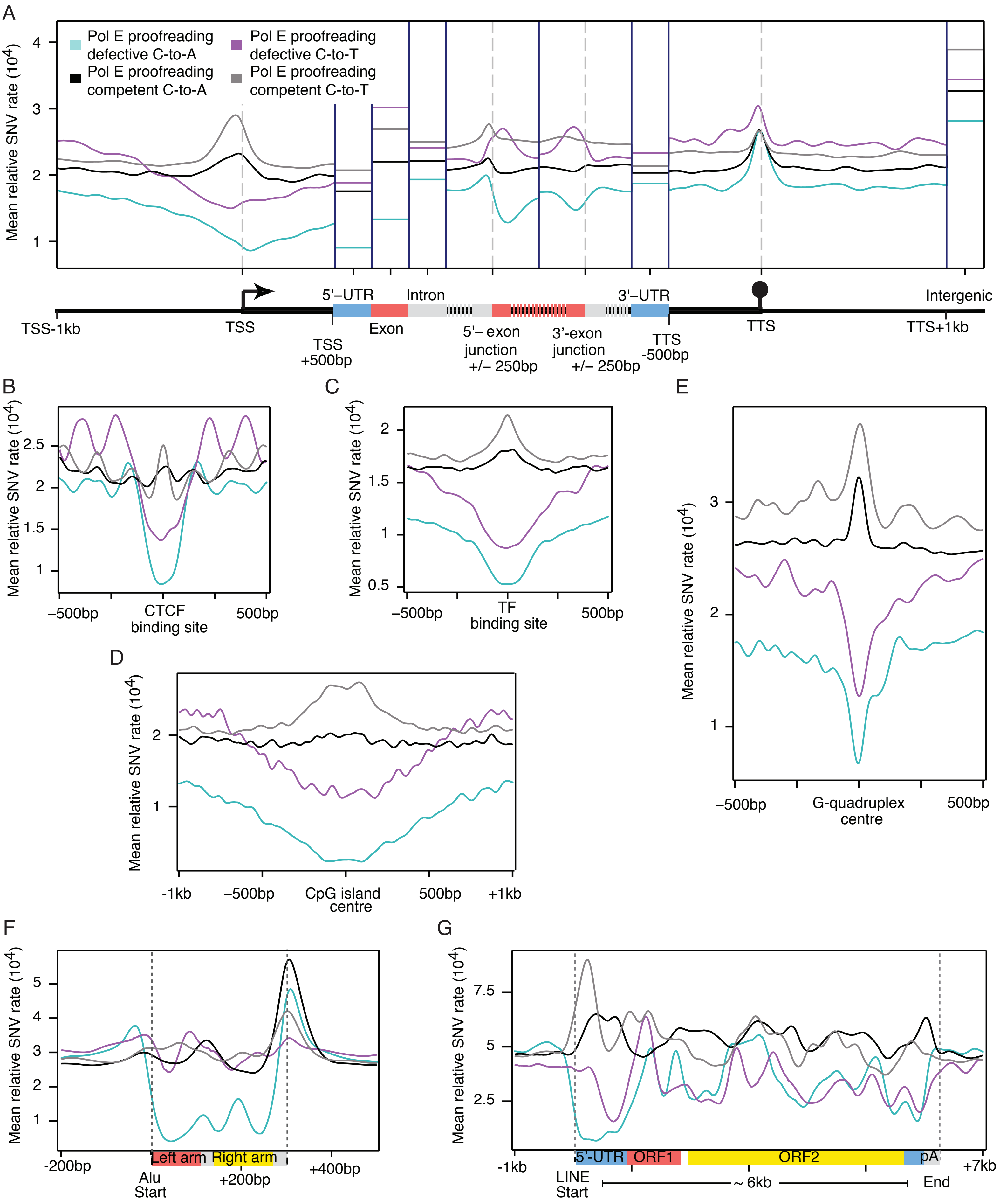
A
Pol E proofreading defective C-to-A
Pol E proofreading competent C-to-A
Pol E proofreading defective C-to-T
Pol E proofreading competent C-to-T
Mean relative SNV rate (10^4)
TSS-1kb
TSS
TSS +500bp
5'–UTR
Exon
Intron
5'– exon junction +/– 250bp
3'-exon junction +/– 250bp
3'–UTR
TTS -500bp
TTS
Intergenic
TTS+1kb
B
CTCF binding site
–500bp
500bp
C
TF binding site
D
CpG island centre
-1kb
–500bp
500bp
+1kb
E
G-quadruplex centre
F
Alu Start
Left arm
Right arm
-200bp
+200bp
+400bp
G
LINE Start
5'-UTR
ORF1
ORF2
pA
End
~ 6kb
-1kb
+7kb

Fig. S7. SNVs from POLE proofreading deficient cancers follow distinct patterns. **(A)** Metaprofile of SNV rates over ~23,000 protein-coding genes in tumour samples separated into those that are Pol E-proofreading defective (n=8) and to all other tumours (n=2,694). The oxidative damage-dependent SNV profiles in proofreading-defective tumours show similar distributions to AP-sites, whereas the pattern is lost in control tumours. C-to-A SNV distribution patterns are largely mimicked by C-to-T SNV patterns, except for exons, which show an accumulation of C-to-T while C-to-A shows a depletion. **(E-H)** Metaprofiles of SNV rates centred on CTCF-binding sites (n=48,671), transcription factor-binding sites in DHS regions (n=253,613), CpG islands (n=27,443), and G-quadruplex structures (n= 359,449). SNV profiles in proofreading defective tumours mimic the damage profiles. **(I, J)** Metaprofiles across 848,350 *Alu* and 2,533 *LINE* elements. SNV rates in proofreading defective tumours are reduced compared with damage profiles. C-to-T SNVs at large follow the C-to-A SNV profiles, except for *Alu* elements, where mutations are not reduced relative to the flanking sequence, and exons, where it even accumulates.

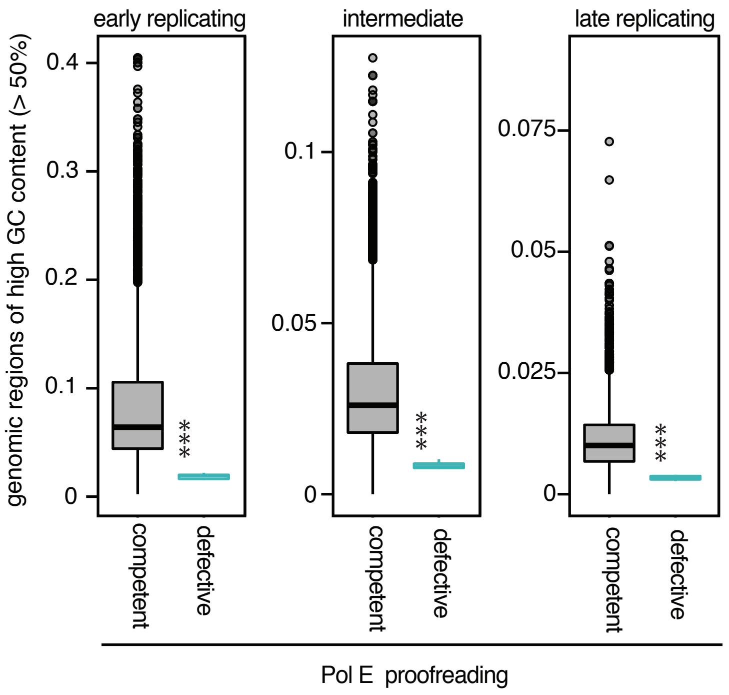

Fig. S8. SNVs from POLE proofreading deficient cancers spare out high GC content DNA, irrespective of replication timing.

Tumours that are proofreading defective display lower proportions of SNVs in GC-rich regions irrespective of whether these GC-rich regions localise into early, intermediate, or late replicating DNA. Mutation rates in high GC content DNA are assessed separately for early, intermediate, and late replicating DNA, as determined from Repli-Seq data in HepG2 cells. 1kb bins of the genome are separated into equally sized tertiles of replication timing. Boxplots are depicted for C-to-A SNVs (including the reverse complement G-to-T) in genomic regions of high GC content (>50%). Due to bias of high GC content distribution and replication timing, high GC content DNA is overrepresented in early replicating DNA (207Mb) versus intermediate (78Mb) and late replicating DNA (24Mb). Therefore, all three groups were assessed separately. Tumour samples are separated into those that are Pol E-proofreading defective (n=8) and to all other tumours (n=2,694). Asterisks indicate significance of $p<0.001$ by Wilcoxon rank test comparing the PolE proofreading deficient to competent tumours.

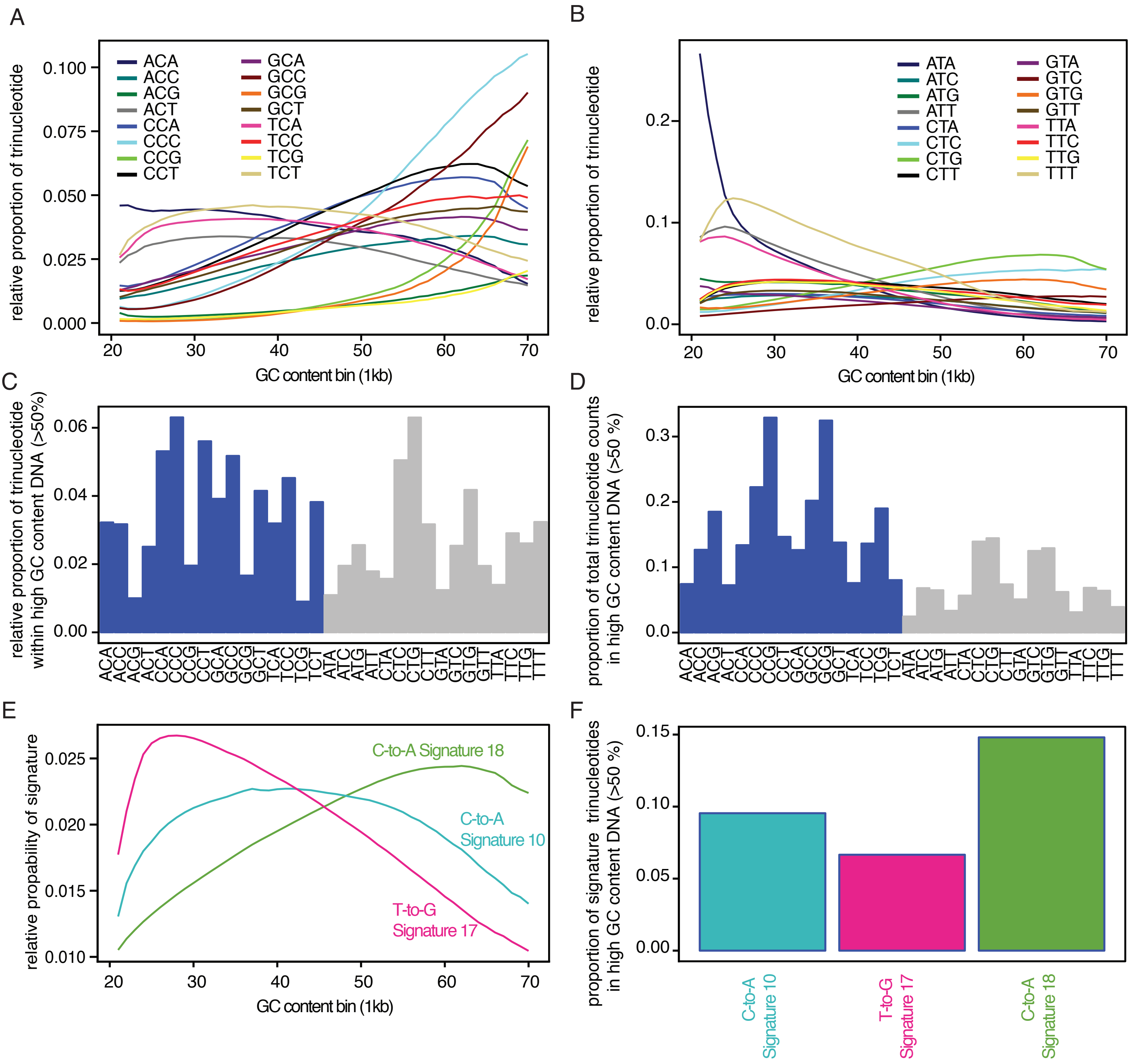

Fig. S9. Trinucleotide distribution in high GC content DNA. **(A&B)** Relative trinucleotide proportions of G/C centered trinucleotides (A) and A/T centered trinucleotides (B) per GC content category. Trinucleotides were counted in GC content categories in 1kb bins. Reverse complement trinucleotides were combined and assessed as the relative proportion within each GC content category. **(C)** Relative trinucleotide proportions in GC content >50% GC content were combined and plotted as the relative proportion of trinucleotides within GC content >50%. **(D)** Trinucleotide proportion in GC content >50% versus GC content <50%. The proportion was assessed as the counts of each trinucleotide falling into GC content >50% versus the total counts of trinucleotide in the genome. **(E)** Relative distribution of signature relevant trinucleotides over GC content categories. Trinucleotides were summed in proportion to their contribution to C-to-A mutations in Signature 18, C-to-A mutations in Signature 10, and T-to-G mutations in Signature 17. **(F)** Proportion of trinucleotides in high GC content DNA (>50%) dependent on their contribution to signatures. This represents the proportion of mutations from a particular signature expected to fall into high GC content DNA (>50%) based in sequence content alone.
