# Supplementary material for: Genomic landscape of oxidative DNA damage and repair reveals regioselective protection from mutagenesis
Source: Genome Biol. 2018 Dec 7;19:215. doi: 10.1186/s13059-018-1582-2 (PMC6284305; doi:10.1186/s13059-018-1582-2)
Supplement: Supplementary file 2 — Table S1. Oligonucleotides and primers used for in vitro pulldown experiments. Table S2. Selected tumor samples with coding mutations in OGG1, APEX1, of FEN1. Table S3. Selected tumor samples with polymerase epsilon proofreading defect. (DOCX 17 kb) [file 13059_2018_1582_MOESM2_ESM.docx]

Table S1: Oligonucleotides and primers used for in-vitro pulldown experiments.

| Oligo name | Sequence |
| --- | --- |
| guanine-forward | 5’-P-GTAAAACGACGGCCAGAGCTTACTCTAGAAGAATTCTCACTCT TT-**G-**TTTCTCACTGGATCCACAGATATCACACAGTCATAGCTGTTTC CTG-3’ |
| 8-oxoG-forward | 5’-P-GTAAAACGACGGCCAGAGCTTACTCTAGAAGAATTCTCACTC TTT**-8-oxoG-**TTTCTCACTGGATCCACAGATATCACACAGTCATAGCT GTTTCCTG-3’ |
| guanine -reverse | 5’-P-CAGGAAACAGCTATGACTGTGTGATATCTGTGGATCCAGTGAG AAA-C-AAAGAGTGAGAATTCTTCTAGAGTAAGCTCTGGCCGTCGTT TTAC-3’ |
| M13-forward | 5’- GTAAAACGACGGCCAG-3’ |
| M13-reverse | 5’- CAGGAAACAGCTATGAC-3’ |

Table S2: Selected tumour samples with coding mutations in OGG1, APEX1, of FEN1.

| Sample IDs [tumor_wgs_aliquot_id] | Total C-to-A | Proportion C-to-A | Total mutation count | Cancer type | Coding Mutation | Mutation type | Amino acid change­­ |
| --- | --- | --- | --- | --- | --- | --- | --- |
| 42f88b95-fa12-47c7-93f1-cf72f207291c | 1,308 | 0.16 | 7,945 | Kidney-RCC | OGG1 | frameshift | H273X |
| 4a1ad661-f6ae-44e8-b50b-72ff658ff22b | 1,480 | 0.19 | 7,872 | CNS-GBM | OGG1 | missense | R277H |
| 7456abd5-303e-4e6f-bf4e-47efefc7310f | 913 | 0.16 | 5,729 | Breast-AdenoCA | OGG1 | missense | G290E |
| dc4ba4bc-6333-4fe9-8805-e058cc9e6e18 | 1,963 | 0.17 | 11,509 | Panc-Endocrine | OGG1 | missense | Q226H |
| e6801359-d1d7-4871-b2fb-180674a2e469 | 1,506 | 0.16 | 9,141 | Kidney-RCC | OGG1 | missense | T177A |
| f7e7d61f-e2dc-b523-e040-11ac0c482000 | 477 | 0.16 | 3,011 | Breast-AdenoCA | OGG1 | missense | Q263H |
| fc5dc6d8-62d2-76d8-e040-11ac0d4863c3 | 1,494 | 0.17 | 8,637 | Breast-AdenoCA | OGG1 | stop gain | R340* |
| 45a7949d-e63f-4956-866c-df51257032de | 2,631 | 0.10 | 25,181 | Bladder-TCC | APEX1 | missense | D15H |
| 9ebac79d-8b38-4469-837e-b834725fe6d5 | 2,594 | 0.16 | 16,191 | Panc-AdenoCA | APEX1 | missense | Q137P |
| bf91afc4-aa2b-4365-80c5-b98c9d118e10 | 333 | 0.13 | 2,543 | Panc-Endocrine | APEX1 | missense | S56R |
| 369c06f2-8904-49cb-99d1-dd297ed0cd0c | 3,424 | 0.17 | 20,565 | Lung-SCC | FEN1 | missense | I79M |
| 81b1e78c-6032-4ff4-b52a-83456b9450ea | 9,708 | 0.33 | 29,682 | ColoRect-AdenoCA | FEN1 | missense | R73C |
| f7b84c09-15d4-3046-e040-11ac0c4847ff | 455 | 0.18 | 2,528 | Breast-AdenoCA | FEN1 | missense | E257K­­­ |

Table S3: Selected tumour samples with polymerase epsilon proofreading defect.

| Sample IDs [tumor_wgs_aliquot_id] | Total C-to-A | Proportion C-to-A | Total mutation count | Cancer type | Proportion Signature 10 |
| --- | --- | --- | --- | --- | --- |
| 00aa769d-622c-433e-8a8a-63fb5c41ea42 | 115,337 | 0.46 | 252,195 | ColoRect-AdenoCA | 0.65 |
| 0980e7fd-051d-45e9-9ca6-2baf073da4e8 | 396,377 | 0.44 | 907,411 | ColoRect-AdenoCA | 0.60 |
| 14c5b81d-da49-4db1-9834-77711c2b1d38 | 989,958 | 0.40 | 2,502,427 | ColoRect-AdenoCA | 0.57 |
| 154f80bd-984c-4792-bb89-20c4da0c08e0 | 126,870 | 0.45 | 280,527 | ColoRect-AdenoCA | 0.63 |
| 2df02f2b-9f1c-4249-b3b4-b03079cd97d9 | 964,307 | 0.38 | 2,570,161 | ColoRect-AdenoCA | 0.41 |
| 6ca5c1bb-275b-4d05-948a-3c6c7d03fab9 | 243,272 | 0.28 | 871,206 | ColoRect-AdenoCA | 0.55 |
| 93ff786e-0165-4b02-8d27-806d422e93fc | 436,686 | 0.43 | 1,024,918 | ColoRect-AdenoCA | 0.50 |
| b0a83df8-dd2c-4c1b-b238-9081d2c22258 | 163,724 | 0.54 | 303,201 | Uterus-AdenoCA | 0.65 |
